# Analysis of Bioactive Components in the Fruit, Roots, and Leaves of *Alpinia oxyphylla* by UPLC-MS/MS

**DOI:** 10.1155/2021/5592518

**Published:** 2021-07-09

**Authors:** Li Ying, Deli Wang, Guankui Du

**Affiliations:** ^1^Key Laboratory of Molecular Biology, Hainan Medical University, Haikou, China; ^2^Haikou Customs, District P. R. China, Haikou, China; ^3^Hainan-branch Institute of Medicinal Plant Development, Chinese Academy of Medical Sciences & Peking Union Medical College, Haikou 570311, China; ^4^Department of Biochemistry and Molecular Biology, Hainan Medical University, Haikou, China

## Abstract

*Alpinia oxyphylla* (*A. oxyphylla*) fruit has long been used in traditional Chinese medicine. In our study, the bioactive components of its roots, fruit, and leaves were investigated, and their potential medical value was predicted. The root, fruit, and leaf samples were analyzed using a UPLC-MS/MS system. The mass spectrometry outcomes were annotated by MULTIAQUANT. The “compound-disease targets” were used to construct a pharmacology network. A total of 293, 277, and 251 components were identified in the roots, fruit, and leaves, respectively. The fruit of *A. oxyphylla* had a higher abundance of flavonols. The roots of *A. oxyphylla* were enriched in flavonols and phenolic acids. The leaves of *A. oxyphylla* exhibited high contents of flavonols, phenolic acids, and tannins. Furthermore, network pharmacology analysis showed that flavonoids are the most important effectors in the fruit of *A. oxyphylla* and phenolic acids are the most important effectors in the roots and leaves. Moreover, the results suggested that the tissues of *A. oxyphylla* might play a role in the regulation of disease-related genes. The whole plant of *A. oxyphylla* is rich in natural drug components, and each tissue has high medicinal value. Therefore, comprehensive utilization of *A. oxyphylla* can greatly improve its economic value.

## 1. Introduction

*Alpinia oxyphylla (A. oxyphylla)* is commonly used in traditional Chinese medicine (TCM). The dried, ripe fruit of *A. oxyphylla* has long been used for treating diarrhea, enuresis, dementia, and other disorders [1]. Modern pharmacological studies have shown that *A. oxyphylla* extracts have antioxidant and anti-inflammatory capacities [2, 3]. In addition, *A. oxyphylla* has been used for the treatment of diabetes [3] and Alzheimer's disease [4].

Numerous chemical constituents, including flavonoids, diarylheptanoids, sesquiterpenes, sterols, and their glycosides, have been isolated from *A. oxyphylla* [1]. The main flavonoids were chrysin, tectochrysin, izalpinin, and kaempferol [5–7]. Yakuchinone A, yakuchinone B, oxyphyllacinol, and neonootkatol were the main diarylheptanoids [6]. The sesquiterpene constituents, including oxyphyllol A–C, nootkatone, and isocyperol, were extracted by aqueous 80% acetone [8]. The norsesquiterpenes, including oxyphyllenodiol A, oxyphyllenodiol B, oxyphyllenone A, oxyphyllenone B, oxyphyllone E, and oxyphyllone F, have been previously reported [9]. Several steroids have been isolated, such as *β*-sitosterol, stigmasterol, and *β*-daucosterol [10]. These results highlight that *A. oxyphylla* fruit has a variety of drug components, and it is still meaningful to comprehensively determine the chemical components of *A. oxyphylla* tissues.

Liquid chromatography-tandem mass spectrometry (LC-MS/MS) provides a vital tool to systematically analyze TCM metabolites [11]. Two flavonoids (chrysin and tectochrysin) from *A. oxyphylla* fruit extract were determined by LC-MS/MS with a method exhibiting accuracy ranging from −8.8% to 7.5% [5]. Li et al. identified nine compounds from *A. oxyphylla* fruit, which was achieved with 70% ethanol [11]. Moreover, Chen et al. detected the differential secondary metabolites of seed and fruit capsules by LC-MS/MS [12]. Therefore, technical advances in the large-scale analysis of metabolites have helped to reveal the complex processes associated with modulating plant metabolism.

Among the plants of the genus *Alpinia*, the fruit or roots of plants are used as a medicine. Traditionally, the roots and fruit of *Alpinia officinarum* are used as medicines [13]. However, there are few systematic studies on the components of the roots and leaves of *A. oxyphylla*. As a result, the medicinal value of *A. oxyphylla* may be underestimated. Therefore, the roots, fruit, and leaves of *A.* o*xyphylla* were collected, and untargeted metabolomics analysis was performed by LC-MS/MS. Furthermore, network pharmacology analysis can help us comprehensively understand the medicinal value of *A. oxyphylla* tissues.

## 2. Methods

### 2.1. Plant Materials

Fresh *A. oxyphylla* samples were harvested in June 2020 from four-year-old cultivated *A. oxyphylla* plants grown in an experimental plot of the Hainan Branch of the China Pharmaceutical Research Institute, Haikou, China.

### 2.2. Metabolite Extraction

Fresh samples were freeze-dried under vacuum and then ground (30 Hz, 1.5 min) to powder with a grinder (mm 400, Retsch, Germany). One hundred milligrams of powder was dissolved in 1.0 mL of a 70% methanol aqueous solution. The dissolved sample was refrigerated overnight at 4°C three times. The samples were centrifuged at 10,000x g for 10 min at 4°C, and the supernatants were collected and then filtered with a microporous membrane filter (0.22-*μ*m pore size). The prepared extracts were stored in sampler vials for LC-MS/MS analysis.

### 2.3. Untargeted Metabolomics Analysis

All samples were analyzed using an ultraperformance liquid chromatography (UPLC, Shim-pack UFLC SHIMADZU CBM30A)-tandem mass spectrometry (MS/MS, Applied Biosystems 4500 QTRAP) system. First, separation was achieved on a Waters ACQUITY UPLC HSS T3 C18 column (2.1 mm × 100 mm, 1.8-*μ*m particle size) using the UPLC system (Waters, Herts, UK). The column oven was maintained at 40°C, and the flow rate was set at 0.4 mL/min. The mobile phase was composed of solvent A (water with 0.04% acetic acid) and solvent B (acetonitrile with 0.04% acetic acid). Gradient elution conditions were set as follows: 0 min, 95 : 5 V/V (A/B); 11.0 min, 5 : 95 V/V; 12.0 min, 5 : 95 V/V; 12.1 min, 95 : 5 V/V; and 15.0 min, 95 : 5 V/V.

High-resolution MS/MS was used to detect metabolites eluted from the column. The electrospray ionization temperature was set at 550°C, and the MS voltage was set at 5500 V. The curtain gas was set at 25 psi. The collision-activated dissociation was set at high.

To compare the differences in the metabolites, the mass spectral peaks of each metabolite detected in different samples were corrected to ensure the accuracy of qualitative and quantitative analyses.  Figure S1 shows the integral correction results of the quantitative analysis of randomly selected metabolites in different samples. The abscissa is the retention time (min) of the metabolite, and the ordinate is the ion current intensity of metabolite ion detection. The metabolites were quantified by the multiple reaction monitoring (MRM) mode of triple-quadrupole mass spectrometry [14]. Quality control samples were prepared by mixing sample extracts and analyzing the repeatability of samples by the same treatment methods. In the process of instrumental analysis, a quality control sample was analyzed every ten samples to monitor the repeatability of the UPLC-MS/MS system over the entire detection process.

### 2.4. Bioinformatics Dataset of Untargeted Metabolism (for TCM)

Raw UPLC-MS/MS data were processed using the following procedures. For each sample, a matrix of molecular features, such as the retention time and mass-to-charge ratio (m/z), was generated using Analyst 1.6.3 software with default parameters. The structures of metabolites were analyzed with reference to MASSBANK (http://www.massbank.jp/), KNAPSAcK (http://kanaya.naist.jp/KNApSAcK/), HMDB (http://www.hmdb.ca/) [15], MoTo DB (http://www.ab.wur.nl/moto/), and METLIN (http://metlin.scripps.edu/index.php) [16].

After obtaining the mass spectrometric data of metabolites from different samples, the peak area of all mass spectral peaks was integrated, and the peaks of the same metabolite in different samples were integrated and corrected [14].

The mass spectrometry file of each sample was opened with MULTIAQUANT software, and the integration and correction of chromatographic peaks were conducted. The peak area of each chromatographic peak represents the relative levels of the corresponding substances.

### 2.5. Target Identification and Network Construction

The target compounds were searched against the SWISSADME (http://www.swissadme.ch/) [17] and TargetNet (http://targetnet.scbdd.com/calcnet/index/) databases [18], which are designed to identify potential target compounds via various prediction algorithms. *Homo sapiens* origin targets were used in the following analysis. Only targets with 95% possibility were included for the disease-related targets.

To compile the disease targets for susceptibility to atherosclerosis, Alzheimer's disease, liver disease, diabetes mellitus, allergies, Parkinson's disease, and depression, we searched the GeneCards database [19]. For each disease, duplicated targets were removed. The intersection between the drug and disease targets was determined to screen key targets.

The “compound-disease targets” were the intersection of *A. oxyphylla* compound targets and disease targets. The network was constructed and analyzed with the Cytoscape platform [20].

### 2.6. Systematic Correlativity Analysis and Statistical Analysis

Pearson's correlation, one-way analysis of variance (ANOVA), and hierarchical (average linkage) clustering were conducted for the untargeted metabolism analyses. *P*-values of the ANOVA were adjusted for the false discovery rate. Principal component analysis (PCA) and partial least squares discrimination analysis (PLS-DA) of the metabolites were performed using SIMCA v14.0 (Umetrics, Umea, Sweden).

## 3. Results

### 3.1. Untargeted Metabolite Profiling of the Metabolites in Different Tissues

**Figure 1:**
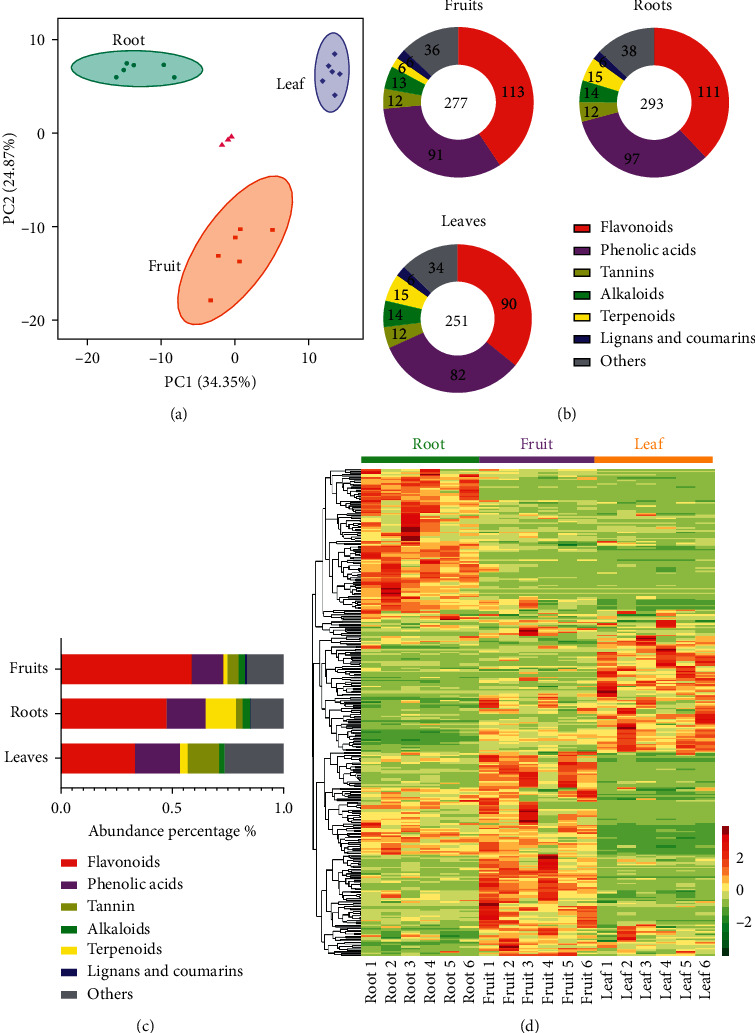
Untargeted metabolite profiling identified the metabolites in tissues of *A. oxyphylla*. (a) PCA data of the samples from three different tissues. Green spots indicate samples from the roots, purple spots indicate samples from the fruit, and yellow spots indicate samples from the leaves. (b) These differentially accumulated metabolites were assigned to various secondary metabolic categories. (c) Percentages of different kinds of metabolites. (d) A heatmap of the relative amounts of differentially accumulated metabolites from the three different plant tissues. The heatmap scale ranges from −1 to +1 after data homogenization.

A total of 312 secondary metabolites were found by untargeted metabolomics analysis (Table S1), including phenolic acids, flavonols, tannins, lignans, coumarins, terpenoids, alkaloids, and quinones. PCA data showed three distinct sample groups, indicating that there was separation among the three tissues (Figure 1(a)). As shown in Figure 1(b), the roots, fruit, and leaves contained 293, 277, and 251 metabolites, respectively. In total, the abundance of metabolites in the roots and fruit was not significantly different, while the abundance of metabolites in the leaves was approximately 51.41% of that in the fruit. All annotated metabolites were classified to identify the differentially accumulated metabolites between tissues (Figure 1(b)). In the roots, 111 flavonoids accounted for 47.40% of the total abundance, 97 phenolic acids accounted for 17.51%, and 15 terpenoids accounted for 13.70% (Figures 1(b) and 1(c)). Among the fruits, flavonoids were the most abundant, with 113 species in total, accounting for 58.86% of the total abundance. The abundance of phenolic acids ranked second, with 91 species, accounting for 14.09%. The abundance of tannins was the third highest, with 13 species, accounting for 5.00% (Figures 1(b) and 1(c)). In the leaves, the three compounds with the highest contents were flavonoids (90 species, accounting for 33.50%), phenolic acids (82 species, accounting for 20.08%), and tannic acids (12 species, accounting for 13.91%) (Figures 1(b) and 1(c)). Moreover, 116 metabolites predominantly accumulated in the roots, 120 metabolites were present at relatively high abundance in the fruits, and 76 metabolites were more highly distributed in the leaves (Figure 1(d)). Therefore, the characteristics of metabolites in the fruit, roots, and leaves of *A. oxyphylla* were significantly different.

### 3.2. Variations in the Abundance Levels of Flavonoids among Tissues

**Figure 2:**
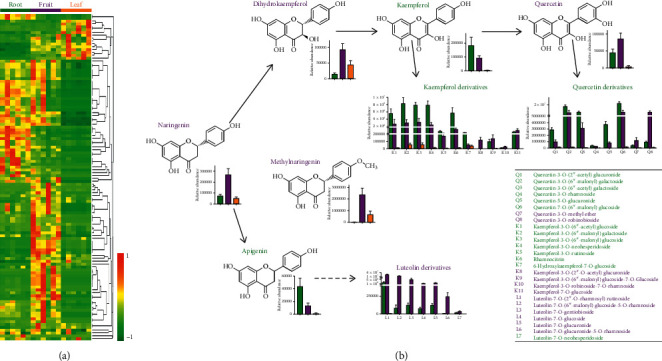
Accumulation of flavonoids in the three tissues. (a) The heatmap scale ranges from −1 to +1 after data homogenization. (b) The biosynthetic pathway of flavonoids. The green color text indicates that the relative concentration was higher in the roots than in the other tissues, the purple color text indicates that the relative concentration was higher in the fruit, and the yellow color text indicates that the relative concentration was higher in the leaves.

As shown in Figure 2, 115 flavonoids were identified in *A. oxyphylla* tissues. Heatmap clustering analysis found that more flavonoids accumulated in the fruit than in the roots and leaves (Figure 2(a)). The phenolic acids with the highest abundance in the fruit were prunetin, rhamnetin, and luteolin-7-O-glucuronide-5-O-rhamnoside. The 3 most abundant phenolic acids in the roots were delphinidin-3-O-(6”-O-*p*-coumaroyl) glucoside, hyperin, and quercetin-7-O-(6”-malonyl) glucoside. The phenolic acids with the highest abundance in the leaves were pinostrobin, epicatechin glucoside, and catechin-catechin-catechin.

Furthermore, the metabolites were assigned to multiple synthetic pathways of flavonoids (Figure 2(b)). Naringenin, dihydrokaempferol, quercetin, methylnaringenin, two quercetin derivatives, four kaempferol derivatives, and six luteolin derivatives were highly accumulated in the fruit. Kaempferol, kaempferol derivatives, six quercetin derivatives, and seven kaempferol derivatives were highly accumulated in the roots. Therefore, the fruit, roots, and leaves of *A. oxyphylla* might adopt different pathways to synthesize flavonoids, resulting in different dominant flavonoids in these tissues.

### 3.3. Variations in the Abundance Levels of Phenolic Acids among Tissues

**Figure 3:**
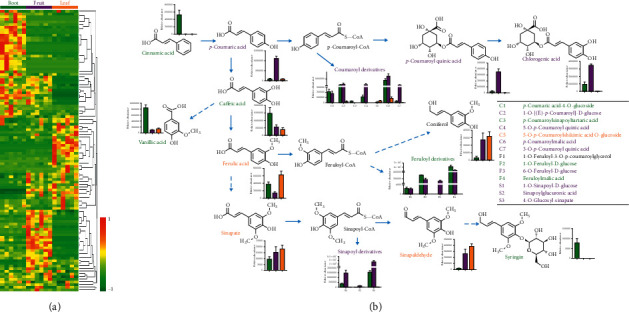
Accumulation of phenolic acids in the three tissues. (a) The heatmap scale ranges from −1 to +1 after data homogenization. (b) The biosynthetic pathway of phenolic acid. The green color text indicates that the relative concentration was higher in the roots than in the other tissues, the purple color text indicates that the relative concentration was higher in the fruit, and the yellow color text indicates that the relative concentration was higher in the leaves.

As shown in Figure 3, a large number of phenolic acids accumulated in the roots, fruit, and leaves (Figure 3(a)). Moreover, the dominant phenolic acids in the fruit, roots, and leaves of *A. oxyphylla* were quite different. 3,4,5-Trimethoxyphenyl-1-O-glucoside, 4-O-glucosyl-sinapate, and dibutyl phthalate were the 3 most abundant phenolic acids in the fruit. 3,4,5-Trimethoxyphenyl-1-O-glucoside, 1,7-bis (4-hydroxy-3-methoxyphenyl) hepta-4,6-dien-3-one, and feruloylmalic acid were the most abundant phenolic acids in the roots. 1,7-Bis(4-hydroxy-3-methoxyphenyl) hepta-4,6-dien-3-one, dibutyl phthalate, and vanillin were the 3 most abundant phenolic acids in the leaves.

Then, phenolic acids were enriched in known synthetic pathways (Figure 3(b)). Cinnamic acid, caffeic acid, vanillic acid, four coumaroyl derivatives, and three feruloyl derivatives were highly accumulated in the roots. *p*-Coumaric acid, *p*-coumaroyl, quinic acid, chlorogenic acid, coniferol, four coumaroyl derivatives, two feruloyl derivatives, and three sinapoyl derivatives were highly accumulated in the fruit. Ferulic acid, coniferol, sinapate, and sinapaldehyde were highly accumulated in the leaves. Therefore, different phenolic acid synthetic strategies were employed in the fruit, roots, and leaves of *A. oxyphylla*.

### 3.4. Network Pharmacology Analysis Based on Major Components in Tissues

**Figure 4:**
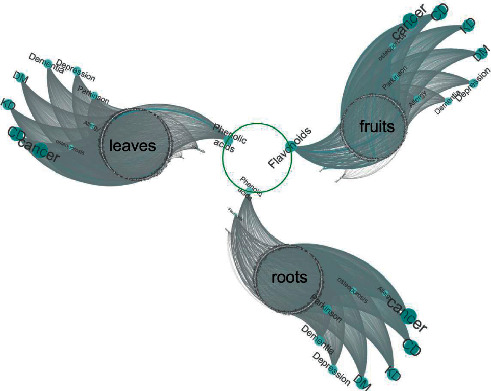
Comprehensive representation of the built network of bioactive constituents in *Alpinia oxyphylla* and diseases.

In fruits, the 20 most abundant metabolites accounted for 52.90% of the total, of which 13 flavonoids accounted for 36.77% (Table 1). These compounds were accepted as candidates to predict the targets. The GeneCards database was used to predict the disease (cancer, osteoporosis, allergic disease, dementia, Parkinson's disease, kidney disease, diabetes mellitus, cardiovascular disease, and depression) targets. Three hundred fourteen overlapping genes were selected as potential targets for integrative network analysis. Thirteen flavonoids had 184 target genes, while phenolic acids had 44 targets. Network pharmacology analysis showed that flavonoids are the main effectors, which could interfere not only with cancer, cardiovascular disease, kidney diseases, and diabetes mellitus but also with depression (Figure 4).

In the roots, among the 20 most abundant metabolites, there were 9 flavonoids (27.5%), 4 phenolic acids (9.52%), and 4 terpenes (8.46%) (Table 2). In addition, a total of 379 genes were obtained for integration network analysis. Among them, phenolic acids may be involved in the regulation of 190 genes, flavonoids are associated with 126 genes, and terpenoids may target 34 genes. Network pharmacology analysis showed that phenolic acid is the main effective component of the roots, and it has the potential to interfere with diseases such as cancer, cardiovascular disease, kidney disease, diabetes, depression, dementia, and Parkinson's disease (Figure 4).

In leaves, the top 20 metabolites accounted for 65.79%, and the highest contents were observed for flavonoids (6 species, accounting for 23.99%), tannins (5 species, accounting for 11.53%), and phenolic acid compounds (3 types, accounting for 7.86%) (Table 3). Furthermore, 418 target genes were found, among which phenolic acids target 305 genes and flavonoids may affect the expression of 137 genes. Therefore, phenolic acids may also be the main active components in leaves (Figure 4). Therefore, the roots, leaves, and fruit of *A. oxyphylla* can be used as a candidate component source to intervene in multiple diseases.

## 4. Discussion

Multiple bioactive components have been separated from *A. oxyphylla* fruit [8]. In the present study, we analyzed the chemical components in the roots, leaves, and fruit of *A. oxyphylla* through UPLC-MS/MS. Based on the analysis results, the intervention effects of different tissues of *A. oxyphylla* on multiple diseases were predicted.

In this study, PCA showed that the components in fruits, roots, and leaves were quite different. The metabolome data were further analyzed by orthogonal partial least squares discriminant analysis (OPLS-DA), which further demonstrated the differences among the fruit, roots, and leaves [21]. Permutation verification of OPLS-DA (*n* = 200, 200 permutation experiments) showed that the R2' and Q2' were both smaller than the R2 and Q2 of the original model, and this model was meaningful (Figure S2).

Previous studies have separated hundreds of essential oil components and 128 other types of components from the fruit of *A. oxyphylla*, including 81 terpenes, six diarylheptanoids, seven flavonoids, and five steroids [1]. These studies used different methods to extract the fruit of *A. oxyphylla* and obtained a variety of components. In addition, due to the different production areas of *A. oxyphylla*, the components were also affected [22]. In this study, 70% methanol was used for extraction, and a total of 277 secondary metabolites were obtained in the fruit of *A. oxyphylla*. Some components have been reported in previous studies, such as kaempferol, while a number of components were highlighted here for the first time. Moreover, the relative abundance of each component was also quantified, which provided a basis for the functional prediction of *A. oxyphylla* fruit.

Studies have shown that multiple flavonoids and phenolic acids were isolated from *A. oxyphylla* fruit [7, 23–25]. The isolated flavonoids included tectochrysin, izalpinin, kaempferide, kaempferol-7,4-dimethyl ether, chrysin, rhamnocitrin, and pinocembrin [7, 23, 24]. The isolated phenolic acids included protocatechuic acid, vanillic acid, 3,5-dihydroxy-4-methoxybenzoic acid, and isovanillin [25]. In the present study, the analysis results showed that the flavonoids and phenolic acids in the fruit were the main components, accounting for 72.95% of the total abundance. Among flavonoids, prunetin, rhamnetin, pinostrobin, oxyphyllol D, pinocembrin, gingerenone A, luteolin derivatives, and quercetin derivatives were the dominant flavonoids. A variety of kaempferol derivatives have also been found. Among phenolic acids, the abundance of sinapoyl derivatives, coumaroyl derivatives, and *p*-coumaric acid was relatively high. Therefore, flavonoids and phenolic acids are the characteristic components of *A. oxyphylla* fruit and can reflect its medicinal value.

Recent studies have shown that *A. oxyphylla* fruit can delay heart aging [26], provide neuroprotection in Alzheimer's disease [4], enhance kidney function [27], and induce cancer cell apoptosis [28]. Moreover, prunetin could induce cell death in gastric cancer cells, relax aortic rings, and promote bone regeneration [29–31]. Rhamnetin could play a role in inducing cancer cell apoptosis, inhibiting cell proliferation, and preventing cancer formation [32–34]. Rhamnetin has the potential to treat oxidative myocardial disease [35]. Pinostrobin may serve as a novel agent for lipid management, cancer treatment, and Parkinson's disease neuroprotection [36–38]. Pinocembrin is effective in treating ischemic stroke, and it also shows excellent neuroprotective potential [39]. Gingerenone A may be used as a potential therapeutic candidate for the treatment of obesity and diabetes [40, 41]. Studies have shown that kaempferol has multiple bioactivities, such as antioxidant, neuroprotective, anticancer, anti-inflammatory, antidiabetic, and antiosteoporotic activities [42, 43]. Phenolic acids have many unique functions, such as memory improvement, antioxidation, antidiabetic, anti-inflammation, and antiaging functions [44–46]. In the present study, the 20 most abundant components in fruit could dock to 314 genes, which were categorized into various pathways, such as respiratory electron transport, LPA receptor-mediated events, and the HIF-1-alpha transcription factor network (Table S2). Network pharmacology predictions showed that the components and targets were associated with cancer, cardiovascular disease, kidney diseases, diabetes mellitus, and depression. These results match those observed in previous studies showing that *A. oxyphylla* fruit can play a role in the treatment of a variety of diseases.

The roots of some *Alpinia* species, such as *A. officinarum*, are used for medicine [47]. The main components in the roots of *A. officinarum* were kaempferol, quercetin, diphenylheptane, and volatile oils [47]. *A. officinarum* is used to treat digestive disorders, stomachache, flatulence, and the common cold [47]. However, the medicinal value of *A. oxyphylla* roots is still mostly unknown. In the present study, a large number of secondary metabolites were detected in the roots. Flavonoids and phenolic acids accounted for 64.91% of the total abundance, and terpenoids accounted for 13.70%. Therefore, flavonoids, phenolic acids, and terpenoids are the representative components of *A. oxyphylla* roots.

It has been reported that the physiological activities of quercetin include anticancer, hypoglycemic, and antiobesity activities [42, 43, 48]. Delphinidin has a variety of pharmacological activities, including anticancer, cardiovascular protection, neuroprotection, antidiabetes, and antiobesity activities [49]. Ferulic acid could offer beneficial effects, such as anticancer, antidiabetes, and antineurodegenerative effects [50]. Nootkatol could prevent UV-induced photoaging [51]. In the present study, delphinidin, kaempferol derivatives, and quercetin derivatives were the dominant flavonoid components in *A. oxyphylla* roots, while feruloylmalic acid and feruloyl derivatives were the dominant phenolic acid components. Among terpenoids, oxyphyllol D, oxyphyllol A, oxyphyllenone B, and nootkatol were present at higher levels. Moreover, the top 20 abundant components in the roots docked with 378 genes, which were related to 220 pathways, including lipid metabolism, inflammatory response, and neurotransmitter metabolism (Table S2). The target genes might be involved in multiple diseases, including cancer, cardiovascular disease, kidney diseases, diabetes mellitus, depression, dementia, and Parkinson's disease. These analysis results indicate that *A. oxyphylla* roots also have high medicinal value.

Few studies have analyzed the chemical constituents of volatile oil and organic acids from the leaves of *A. oxyphylla* [52]. Systematically analyzed chemical components of *A. oxyphylla* leaves have not been reported. The present study demonstrated that the total abundance of metabolites in leaves was approximately 51.40% of that in the fruit, and the dominant components were flavonoids (33.50%), phenolic acids (20.08%), and tannins (13.91%). Pinostrobin, epicatechin, rhamnetin, pinocembrin, and prunetin were the most abundant flavonoids. Among the tannins, procyanidin C1, procyanidin B2, procyanidin B3, procyanidin B4, and procyanidin C2 had a high abundance. Therefore, this study systematically analyzed the drug components of *A. oxyphylla* leaves and clarified the main chemical components of *A. oxyphylla* leaves.

Recent studies have shown that epicatechin plays a role in improving cardiovascular and cerebrovascular diseases and exerts anti-inflammatory, antidiabetic, and neuroprotective effects [53]. Procyanidin is considered to be involved in lipid regulation and cancer treatment [54, 55]. In the present study, the top 20 abundant components in the leaves of *A. oxyphylla* docked with 416 genes, which were related to multiple pathways, including respiratory electron transport, IL1-mediated signaling events, and the TNF receptor signaling pathway (Table S2). Network pharmacology predictions showed that components in the leaves were also associated with a variety of diseases. Even its target genes had a higher relationship degree with the analyzed diseases than those of roots and fruits. *A. oxyphylla* leaves are readily available. Thus, the use of leaves as medicine can significantly increase the economic value of *A. oxyphylla*.

In summary, metabolic profiles revealed that the levels of metabolite accumulation might vary significantly among the fruit, roots, and leaves of *A. oxyphylla*. The representative components of *A. oxyphylla* fruit were flavonoids and phenolic acids. Flavonoids, phenolic acids, and terpenoids were the main components in *A. oxyphylla* roots. Flavonoids, phenolic acids, and tannins were the dominant components in *A. oxyphylla* leaves. Furthermore, the network pharmacology predictions suggest that the fruit, roots, and leaves of *A. oxyphylla* were associated with cancer, cardiovascular disease, kidney diseases, and diabetes mellitus. In addition, different tissues of *A. oxyphylla* could be used to treat more different diseases. Therefore, further studies on the drug components and functions of tissues of *A. oxyphylla* will help to improve the medicinal value and economic value of *A. oxyphylla*.

## Figures and Tables

**Table 1:** Top 20 abundant components in fruits.

Rank	Formula	Compounds	Class I	Class II	Mean abundance
1	C_16_H_12_O_5_	Prunetin (5,4'-dihydroxy-7-methoxyisoflavone)	Flavonoids	Isoflavones	47867500
2	C_16_H_12_O_7_	Rhamnetin (7-O-methxyl quercetin)	Flavonoids	Flavonols	36629167
3	C_27_H_28_O_16_	Luteolin (7-O-glucuronide-5-O-rhamnoside)	Flavonoids	Flavonoid	25471383
4	C_21_H_26_O_6_	5-Hydroxy-1,7-bis (4-hydroxy-3-methoxyphenyl) heptan-3-one	Others	Others	25408500
5	C_22_H_30_O_7_	3,5-Dihydroxy-meodah	Others	Others	21946233
6	C_16_H_14_O_4_	Pinostrobin	Flavonoids	Dihydroflavone	19766500
7	C_21_H_20_O_12_	Quercetin-3-O-galactoside (hyperin)	Flavonoids	Flavonols	16930800
8	C_21_H_20_O_11_	Luteolin-7-O-glucoside (cynaroside)	Flavonoids	Flavonoid	14381533
9	C_22_H_28_O_7_	5-Hydroxy-1-(4-hydroxy-3,5-dimethoxyphenyl)-7-(4-hydroxy-3-methoxyphenyl) heptan-3-one	Others	Others	12284683
10	C_21_H_20_O_12_	Quercetin-3-O-glucoside (isoquercitrin)	Flavonoids	Flavonols	12108717
11	C_15_H_24_O_3_	Oxyphyllol D	Terpenoids	Sesquiterpenoids	11446750
12	C_15_H_12_O_4_	Pinocembrin (dihydrochrysin)	Flavonoids	Dihydroflavone	11027850
13	C_45_H_38_O_18_	Catechin-catechin-catechin	Flavonoids	Flavanols	10100900
14	C_22_H_24_O_11_	Hesperetin-5-O-glucoside	Flavonoids	Dihydroflavonol	10053150
15	C_12_H_16_O_6_	2-Hydroxy-3-carboxy-4-linyldihydroxy	Others	Others	9552900
16	C_27_H_30_O_16_	Quercetin-3-O-robinobioside	Flavonoids	Flavonols	9517433
17	C_21_H_24_O_5_	Gingerenone A	Others	Others	9503050
18	C_24_H_22_O_15_	Quercetin-7-O-(6”-malonyl) glucoside	Flavonoids	Flavonols	9290433
19	C_24_H_22_O_15_	Quercetin-3-O-(6”-malonyl) galactoside	Flavonoids	Flavonols	9010300
20	C_15_H_22_O_8_	3,4,5-Trimethoxyphenyl-1-O-glucoside	Phenolic acids	Phenolic acids	8675383

**Table 2:** Top 20 abundant components in roots.

Rank	Formula	Compounds	Class I	Class II	Mean abundance
1	C_30_H_27_O_14_	Delphinidin-3-O-(6”-O-*p*-coumaroyl)glucoside	Flavonoids	Anthocyanins	40513500
2	C_21_H_20_O_12_	Quercetin-3-O-galactoside (Hyperin)	Flavonoids	Flavonols	23952000
3	C_24_H_22_O_15_	Quercetin-7-O-(6”-malonyl) glucoside	Flavonoids	Flavonols	22008667
4	C_15_H_24_O_3_	Oxyphyllol D	Terpenoids	Sesquiterpenoids	21040167
5	C_20_H_22_O_4_	1-(4-Hydroxy-3-methoxyphenyl)-7-phenyl-3,5-diheptanone	Others	Others	19360450
6	C_24_H_22_O_15_	Quercetin-3-O-(6”-malonyl) galactoside	Flavonoids	Flavonols	17750167
7	C_21_H_20_O_12_	Quercetin-3-O-glucoside (Isoquercitrin)	Flavonoids	Flavonols	17015000
8	C_15_H_22_O_8_	3,4,5-Trimethoxyphenyl-1-O-glucoside	Phenolic acids	Phenolic acids	16549150
9	C_15_H_24_O	Oxyphyllol A	Terpenoids	Sesquiterpenoids	14889410
10	C_21_H_26_O_6_	5-Hydroxy-1,7-bis (4-hydroxy-3-methoxyphenyl) heptan-3-one	Others	Others	14610700
11	C_22_H_24_O_11_	Hesperetin-5-O-glucoside	Flavonoids	Dihydroflavonol	13949667
12	C_21_H_22_O_5_	1,7-Bis(4-hydroxy-3-methoxyphenyl) hepta-4,6-dien-3-one	Phenolic acids	Phenolic acids	13410017
13	C_12_H_18_O_3_	Oxyphyllenone B	Terpenoids	Sesquiterpenoids	12010683
14	C_16_H_12_O_7_	Rhamnetin (7-O-methxyl quercetin)	Flavonoids	Flavonols	11396750
15	C_14_H_14_O_8_	Feruloylmalic acid	Phenolic acids	Phenolic acids	11289450
16	C_20_H_24_O_5_	5-Hydroxy-1-(4-hydroxy-3-methoxyphenyl)-7-(4-hydroxyphenyl) heptan-3-one	Others	Others	11270433
17	C_27_H_30_O_15_	Kaempferol-3-O-neohesperidoside	Flavonoids	Flavonols	9886600
18	C_16_H_20_O_10_	Trihydroxycinnamoylquinic acid	Phenolic acids	Phenolic acids	9825767
19	C_23_H_22_O_13_	Quercetin-3-O-(6”-acetyl) galactoside	Flavonoids	Flavonols	9651967
20	C_15_H_24_O	Nootkatol	Terpenoids	Sesquiterpenoids	9543583

**Table 3:** Top 20 abundant components in leaves.

Rank	Formula	Compounds	Class I	Class II	Mean abundance
1	C_16_H_14_O_4_	Pinostrobin^*∗*^	Flavonoids	Dihydroflavone	28276667
2	C_21_H_26_O_6_	5-Hydroxy-1,7-bis (4-hydroxy-3-methoxyphenyl) heptan-3-one	Others	Others	23139050
3	C_21_H_24_O_11_	Epicatechin glucoside	Flavonoids	Flavanols	12130550
4	C_21_H_26_O_7_	5'-Hydroxyhexahydrocurcumin	Others	Others	11798517
5	C_22_H_30_O_7_	3,5-Dihydroxy-meodah	Others	Others	11779017
6	C_45_H_38_O_18_	Catechin-catechin-catechin^*∗*^	Flavonoids	Flavanols	11665500
7	C_16_H_12_O_7_	Rhamnetin (7-O-methxyl quercetin)	Flavonoids	Flavonols	10039950
8	C_21_H_24_O_5_	Gingerenone A	Others	Others	9896533
9	C_21_H_22_O_5_	1,7-Bis(4-hydroxy-3-methoxyphenyl) hepta-4,6-dien-3-one	Phenolic acids	Phenolic acids	9670050
10	C_16_H_22_O_4_	Dibutyl phthalate	Phenolic acids	Phenolic acids	9167633
11	C_45_H_38_O_18_	Procyanidin C1^*∗*^	Tannins	Proanthocyanidins	8929133
12	C_12_H_16_O_6_	2-Hydroxy-3-carboxy-4-linyldihydroxy	Others	Others	8730317
13	C_30_H_26_O_12_	Procyanidin B2^*∗*^	Tannins	Proanthocyanidins	8077250
14	C_15_H_12_O_4_	Pinocembrin (dihydrochrysin)	Flavonoids	Dihydroflavone	7835000
15	C_30_H_26_O_12_	Procyanidin B3^*∗*^	Tannins	Proanthocyanidins	7340183
16	C_16_H_12_O_5_	Prunetin (5,4'-dihydroxy-7-methoxyisoflavone)^*∗*^	Flavonoids	Isoflavones	7205250
17	C_21_H_22_O_6_	Dihydrocurcumin	Others	Others	6622600
18	C_30_H_26_O_12_	Procyanidin B4^*∗*^	Tannins	Proanthocyanidins	6590183
19	C_8_H_8_O_3_	Vanillin	Phenolic acids	Phenolic acids	6545083
20	C_45_H_38_O_18_	Procyanidin C2^*∗*^	Tannins	Proanthocyanidins	6164967

## Data Availability

All the datasets generated and analyzed during the current study were uploaded with the manuscript as additional files.

## Abbreviations

TCM: Traditional Chinese medicine
LC-MS/MS: Liquid chromatography-tandem mass spectrometry
UPLC: Ultraperformance liquid chromatography
MS/MS: Tandem mass spectrometry
QC: Quality control
ANOVA: Analysis of variance
PCA: Principal component analysis
PLS-DA: Partial least squares discrimination analysis.

## Supplementary Materials

Supplementary Materials Figure S1: MRM metabolite detection. The multipeak diagram shows the substances that were detected in the sample, and each mass spectral peak with different colors represents one detected metabolite. Figure S2: OPLS-DA analysis model verification diagram. Table S1: 312 identified metabolites. Table S2: the target genes were enriched in multiple pathways.

## References

[1] Zhang Q., Zheng Y., Hu X. (2018). Ethnopharmacological uses, phytochemistry, biological activities, and therapeutic applications of *Alpinia oxyphylla Miquel*: a review. *Journal of Ethnopharmacology*.

[2] Xie Y., Xiao M., Li D. (2017). Anti-diabetic effect of *Alpinia oxyphylla* extract on 57BL/KsJ db-/db- mice. *Experimental and Therapeutic Medicine*.

[3] Xie Y., Xiao M., Ni Y. (2018). *Alpinia oxyphylla miq*. extract prevents diabetes in mice by modulating gut microbiota. *Journal of Diabetes Research*.

[4] Xu J., Wang F., Guo J. (2020). Pharmacological mechanisms underlying the neuroprotective effects of *Alpinia oxyphylla miq*. on Alzheimer’s disease. *International Journal of Molecular Sciences*.

[5] Zhao X., Su X., Liu C., Jia Y. (2018). *Alpinia oxyphylla* simultaneous determination of chrysin and tectochrysin from fruits by UPLC-MS/MS and its application to a comparative pharmacokinetic study in normal and dementia rats. *Molecules (Basel, Switzerland)*.

[6] Yuan Y., Tan Y., Xu P. (2014). Izalpinin from fruits of *Alpinia oxyphylla* with antagonistic activity against the rat bladder contractility. *African Journal of Traditional, Complementary and Alternative Medicines*.

[7] Bian Q.-Y., Wang S.-Y., Xu L.-J., Chan C.-O., Mok D. K. W., Chen S.-B. (2013). Two new antioxidant diarylheptanoids from the fruits ofAlpinia oxyphylla. *Journal of Asian Natural Products Research*.

[8] Morikawa T., Matsuda H., Toguchida I., Ueda K., Yoshikawa M. (2002). Absolute stereostructures of three new sesquiterpenes from the fruit of *Alpinia oxyphylla* with inhibitory effects on nitric oxide production and degranulation in RBL-2H3 cells. *Journal of Natural Products*.

[9] Muraoka O., Fujimoto M., Tanabe G. (2001). Absolute stereostructures of novel norcadinane- and trinoreudesmane-type sesquiterpenes with nitric oxide production inhibitory activity from *Alpinia oxyphylla*. *Bioorganic & Medicinal Chemistry Letters*.

[10] Qing Z. J., Yong W., Hui L. Y. (2012). Two new natural products from the fruits of *Alpinia oxyphylla* with inhibitory effects on nitric oxide production in lipopolysaccharide-activated RAW264.7 macrophage cells. *Archives of Pharmacal Research*.

[11] Li Y.-H., Chen F., Wang J.-f., Wang Y., Zhang J.-Q., Guo T. (2013). Analysis of nine compounds from Alpinia oxyphyllafruit at different harvest time using UFLC-MS/MS and an extraction method optimized by orthogonal design. *Chemistry Central Journal*.

[12] Chen F., Li H.-L., Tan Y.-F. (2014). Different accumulation profiles of multiple components between pericarp and seed of *Alpinia oxyphylla* capsular fruit as determined by UFLC-MS/MS. *Molecules*.

[13] Liu X., Hong F. U., Huang H., Chen D., Yao Y. (1999). Identification of *Alpinia officinarum hance* and four confusing species. *Journal of Guangzhou University of Traditional Chinese Medicine*.

[14] Fraga C. G., Clowers B. H., Moore R. J., Zink E. M. (2010). Signature-discovery approach for sample matching of a nerve-agent precursor using liquid chromatography−mass spectrometry, XCMS, and chemometrics. *Analytical Chemistry*.

[15] Wishart D. S., Jewison T., Guo A. C. (2013). HMDB 3.0-the human metabolome database in 2013. *Nucleic Acids Research*.

[16] Montenegro-Burke J. R., Guijas C., Siuzdak G. (2020). METLIN: a tandem mass spectral library of standards. *Computational Methods and Data Analysis for Metabolomics*.

[17] Daina A., Michielin O., Zoete V. (2017). SwissADME: a free web tool to evaluate pharmacokinetics, drug-likeness and medicinal chemistry friendliness of small molecules. *Scientific Reports*.

[18] Yao Z.-J., Dong J., Che Y.-J. (2016). TargetNet: a web service for predicting potential drug-target interaction profiling via multi-target SAR models. *Journal of Computer-Aided Molecular Design*.

[19] Stelzer G., Rosen N., Plaschkes I. (2016). The GeneCards suite: from gene data mining to disease genome sequence analyses. *Current Protocols in Bioinformatics*.

[20] Shannon P., Markiel A., Ozier O. (2003). Cytoscape: a software environment for integrated models of biomolecular interaction networks. *Genome Research*.

[21] Gong L., Chen W., Gao Y. (2013). Genetic analysis of the metabolome exemplified using a rice population. *Proceedings of the National Academy of Sciences*.

[22] Liu H., Han C. R., Liu H. X., Liu Y. F., He M. X. (2008). Study on IR fingerprint spectra of *Alpinia oxyphylla Miq*. *Guang Pu Xue Yu Guang Pu Fen Xi*.

[23] Feng C., Hai-Long L., Yin-Feng T. (2014). Different accumulation profiles of multiple components between pericarp and seed of *Alpinia oxyphylla capsular* fruit as determined by UFLC-MS/MS. *Molecules (Basel, Switzerland)*.

[24] Xie J., Sun B., Wang S., Ito Y. (2009). Isolation and purification of nootkatone from the essential oil of fruits of *Alpinia oxyphylla Miquel* by high-speed counter-current chromatography. *Food Chemistry*.

[25] Xu J., Tan N., Zeng G., Han H., Zhang Y. (2009). Studies on chemical constituents in fruit of *Alpinia oxyphylla*. *China Journal of Chinese Materia Medica*.

[26] Chang Y.-M., Shibu M. A., Chen C.-S. (2021). Adipose derived mesenchymal stem cells along with *Alpinia oxyphylla* extract alleviate mitochondria-mediated cardiac apoptosis in aging models and cardiac function in aging rats. *Journal of Ethnopharmacology*.

[27] Lee Y.-S., Sung Y.-Y., Yuk H. J. (2019). Anti-hyperuricemic effect of *Alpinia oxyphylla* seed extract by enhancing uric acid excretion in the kidney. *Phytomedicine*.

[28] Hui F., Qin X., Zhang Q. (2019). *Alpinia oxyphylla* oil induces apoptosis of hepatocellular carcinoma cells via PI3K/Akt pathway in vitro and in vivo. *Biomedicine & Pharmacotherapy*.

[29] Vetrivel P., Kim S. M., Ha S. E. (2020). Compound prunetin induces cell death in gastric cancer cell with potent anti-proliferative properties: in vitro assay, molecular docking, dynamics, and ADMET studies. *Biomolecules*.

[30] Kim B., Jo C., Choi H. Y., Lee K. (2018). Prunetin relaxed isolated rat aortic rings by blocking calcium channels. *Molecules (Basel, Switzerland)*.

[31] Khan K., Pal S., Yadav M. (2015). Prunetin signals via G-protein-coupled receptor, GPR30(GPER1): stimulation of adenylyl cyclase and cAMP-mediated activation of MAPK signaling induces Runx2 expression in osteoblasts to promote bone regeneration. *The Journal of Nutritional Biochemistry*.

[32] Lan L., Wang Y., Pan Z. (2019). Rhamnetin induces apoptosis in human breast cancer cells via the miR-34a/Notch-1 signaling pathway. *Oncology Letters*.

[33] Jia H., Yang Q., Wang T. (2016). Rhamnetin induces sensitization of hepatocellular carcinoma cells to a small molecular kinase inhibitor or chemotherapeutic agents. *Biochimica et Biophysica Acta (BBA)-General Subjects*.

[34] Ertekin T., Bozkurt O., Eroz R. (2016). May argyrophilic nucleolar organizing region-associated protein synthesis be used for selecting the most reliable dose of drugs such as rhamnetin in cancer treatments?. *Bratisl Lek Listy*.

[35] Li X., Gong J., Meng H. (2016). Investigating molecular mechanisms for rhamnetin in oxidative damaged cardiomyocytes by microarray data analysis. *Minerva Cardioangiologica*.

[36] Li C., Tang B., Feng Y. (2018). Pinostrobin exerts neuroprotective actions in neurotoxin-induced Parkinson’s disease models through Nrf2 induction. *Journal of Agricultural and Food Chemistry*.

[37] Gao W.-Y., Chen P.-Y., Chen S.-F., Wu M.-J., Chang H.-Y., Yen J.-H. (2018). Pinostrobin inhibits proprotein convertase subtilisin/kexin-type 9 (PCSK9) gene expression through the modulation of FoxO3a protein in HepG2 cells. *Journal of Agricultural and Food Chemistry*.

[38] Zaman M., Safdari H. A., Khan A. N. (2019). Interaction of anticancer drug pinostrobin with lysozyme: a biophysical and molecular docking approach. *Journal of Biomolecular Structure and Dynamics*.

[39] Shen X., Liu Y., Luo X., Yang Z. (2019). Advances in biosynthesis, pharmacology, and pharmacokinetics of pinocembrin, a promising natural small-molecule drug. *Molecules (Basel, Switzerland)*.

[40] Suk S., Kwon G. T., Lee E. (2017). Gingerenone A, a polyphenol present in ginger, suppresses obesity and adipose tissue inflammation in high-fat diet-fed mice. *Molecular Nutrition & Food Research*.

[41] Chen J., Sun J., Prinz R. A., Li Y., Xu X. (2018). Gingerenone A sensitizes the insulin receptor and increases glucose uptake by inhibiting the activity of p70 S6 kinase. *Molecular Nutrition & Food Research*.

[42] Kanakis C. D., Tarantilis P. A., Polissiou M. G., Diamantoglou S., Tajmir-Riahi H. A. (2005). DNA interaction with naturally occurring antioxidant flavonoids quercetin, kaempferol, and delphinidin. *Journal of Biomolecular Structure and Dynamics*.

[43] Hämäläinen M., Nieminen R., Vuorela P., Heinonen M., Moilanen E. (2007). Anti-inflammatory effects of flavonoids: genistein, kaempferol, quercetin, and daidzein inhibit STAT-1 and NF-kappaB activations, whereas flavone, isorhamnetin, naringenin, and pelargonidin inhibit only NF-kappaB activation along with their inhibitory effect on iNOS expression and NO production in activated macrophages. *Mediators of Inflammation*.

[44] Kiokias S., Proestos C., Oreopoulou V. (2020). Phenolic acids of plant origin-a review on their antioxidant activity in vitro (O/W emulsion systems) along with their in vivo health biochemical properties. *Foods (Basel, Switzerland)*.

[45] Zhu H., Liang Q.-h., Xiong X.-g. (2018). Anti-inflammatory effects of p-coumaric acid, a natural compound of oldenlandia diffusa, on arthritis model rats. *Evidence-Based Complementary and Alternative Medicine*.

[46] Xiao-ming S., Jia-ying X., Yan W., Yong-cun W., Ning W., Shu-ming C. (2019). Summary on the conditions of ferulic acid esterase and ferulic acid production by microorganism fermentation. *Food Research and Development*.

[47] Pillai M. K., Young D. J., Bin Hj Abdul Majid H. M. (2018). Therapeutic potential of *Alpinia officinarum*. *Mini-Reviews in Medicinal Chemistry*.

[48] Janssen K., Mensink R. P., Cox F. J. (1998). Effects of the flavonoids quercetin and apigenin on hemostasis in healthy volunteers: results from an in vitro and a dietary supplement study. *The American Journal of Clinical Nutrition*.

[49] Chen Z., Zhang R., Shi W. (2019). The multifunctional benefits of naturally occurring delphinidin and its glycosides. *Journal of Agricultural and Food Chemistry*.

[50] de Oliveira Silva E., Batista R. (2017). Ferulic acid and naturally occurring compounds bearing a feruloyl moiety: a review on their structures, occurrence, and potential health benefits. *Comprehensive Reviews in Food Science and Food Safety*.

[51] Woo J. H., Nam D. Y., Kim H. J., Hong P. T. L., Kim W. K., Nam J. H. (2021). Nootkatol prevents ultraviolet radiation-induced photoaging via ORAI1 and TRPV1 inhibition in melanocytes and keratinocytes. *The Korean Journal of Physiology & Pharmacology*.

[52] Niu Q., Gao Y., Liu P. (2020). Optimization of microwave-assisted extraction, antioxidant capacity, and characterization of total flavonoids from the leaves of *Alpinia oxyphylla Miq*. *Preparative Biochemistry & Biotechnology*.

[53] Qu Z., Liu A., Li P. (2021). Advances in physiological functions and mechanisms of (−)-epicatechin. *Critical Reviews in Food Science and Nutrition*.

[54] Downing L. E., Edgar D., Ellison P. A., Ricketts M.-L. (2017). Mechanistic insight into nuclear receptor-mediated regulation of bile acid metabolism and lipid homeostasis by grape seed procyanidin extract (GSPE). *Cell Biochemistry and Function*.

[55] Lee Y. (2017). Cancer chemopreventive potential of procyanidin. *Toxicological Research*.

