# Supplementary material for: Analysis of Bioactive Components in the Fruit, Roots, and Leaves of Alpinia oxyphylla by UPLC-MS/MS
Source: Evid Based Complement Alternat Med. 2021 Jul 9;2021:5592518. doi: 10.1155/2021/5592518 (PMC8286198; doi:10.1155/2021/5592518)
Supplement: Supplementary Materials — Figure S1: MRM metabolite detection. The multipeak diagram shows the substances that were detected in the sample, and each mass spectral peak with different colors represents one detected metabolite. Figure S2: OPLS-DA analysis model verification diagram. Table S1: 312 identified metabolites. Table S2: the target genes were enriched in multiple pathways. [file 5592518.f1.zip › 5592518.f1/Table S1.pdf]

Table S1 312 identified metabolites

RANK	Formula	Compounds	Class I	Class II
1	C16H12O5	Prunetin (5,4'-Dihydroxy-7-methoxyisoflavone)*	Flavonoids	Isoflavones
2	C21H20O12	Quercetin-3-O-galactoside (Hyperin)*	Flavonoids	Flavonols
3	C27H28O16	Luteolin-7-O-glucuronide-5-O-rhamnoside	Flavonoids	Flavonoid
4	C16H12O7	Rhamnetin (7-O-Methxyl Quercetin)	Flavonoids	Flavonols
5	C16H14O4	Pinostrobin*	Flavonoids	Dihydroflavone
6	C21H26O6	5-Hydroxy-1,7-bis(4-hydroxy-3-methoxyphenyl)heptan-3-one	Others	Others
7	C21H20O12	Quercetin-3-O-glucoside (Isoquercitrin)*	Flavonoids	Flavonols
8	C15H24O3	Oxyphyllol D	Terpenoids	Sesquiterpenoids
9	C22H30O7	3,5-Dihydroxy-meodah	Others	Others
10	C24H22O15	Quercetin-7-O-(6"-malonyl)glucoside*	Flavonoids	Flavonols
11	C24H22O15	Quercetin-3-O-(6"-malonyl)galactoside*	Flavonoids	Flavonols
12	C22H24O11	Hesperetin-5-O-glucoside	Flavonoids	Dihydroflavonol
13	C24H22O14	Kaempferol-3-O-(6"-malonyl)glucoside*	Flavonoids	Flavonols
14	C24H22O14	Kaempferol-3-O-(6"-malonyl)galactoside*	Flavonoids	Flavonols
15	C15H12O4	Pinocembrin (Dihydrochrysin)	Flavonoids	Dihydroflavone
16	C7H7NO2	Trigonelline	Alkaloids	Alkaloids
17	C21H20O11	Luteolin-7-O-glucoside (Cynaroside)*	Flavonoids	Flavonoid
18	C16H12O5	Acacetin*	Flavonoids	Flavonoid
19	C30H27O14+	Delphinidin-3-O-(6"-O-p-coumaroyl)glucoside	Flavonoids	Anthocyanins
20	C16H12O5	Genkwanin (Apigenin 7-methyl ether)*	Flavonoids	Flavonoid
21	C17H22O10	4-O-Glucosyl-sinapate*	Phenolic acids	Phenolic acids
22	C45H38O18	Catechin-catechin-catechin*	Flavonoids	Flavanols
23	C21H26O7	5'-Hydroxyhexahydrocurcumin	Others	Others
24	C12H16O6	2-hydroxy-3-carboxy-4-linyldihydroxy	Others	Others
25	C45H38O18	Procyanidin C1*	Tannins	Proanthocyanidins
26	C27H30O16	Quercetin-3-O-robinobioside*	Flavonoids	Flavonols
27	C23H22O12	Kaempferol-3-O-(6"-acetyl)glucoside	Flavonoids	Flavonols
28	C15H22O8	3,4,5-Trimethoxyphenyl-1-O-Glucoside	Phenolic acids	Phenolic acids
29	C27H30O16	Quercetin-3-O-neohesperidoside*	Flavonoids	Flavonols

30	C13H16O9	2,5-Dihydroxybenzoic acid O-glucoside*	Phenolic acids	Phenolic acids
31	C21H24O10	Dihydrocharcone-4'-O-glucoside*	Others	Others
32	C21H24O10	Phloretin-2'-O-glucoside (Phlorizin)*	Flavonoids	Chalcones
33	C45H38O18	Procyanidin C2*	Tannins	Proanthocyanidins
34	C16H22O4	Dibutyl phthalate	Phenolic acids	Phenolic acids
35	C23H22O13	Quercetin-3-O-(6"-acetyl)galactoside	Flavonoids	Flavonols
36	C33H40O20	Quercetin-3-O-(2"-O-Rhamnosyl)rutinoside	Flavonoids	Flavonols
37	C17H14O5	Apigenin-7,4'-dimethyl ether	Flavonoids	Flavonoid
38	C21H24O5	Gingerenone A	Others	Others
39	C13H16O9	Protocatechuic acid-4-O-glucoside*	Phenolic acids	Phenolic acids
40	C25H24O15	Isorhamnetin-3-O-(6"-malonylglucoside)	Flavonoids	Flavonoid
41	C22H22O12	Tricin-4'-methylether-3'-O-glucoside*	Flavonoids	Flavonols
42	C22H22O12	Isorhamnetin-7-O-glucoside (Brassicin)*	Flavonoids	Flavonols
43	C22H22O12	Rhamnetin-3-O-Glucoside*	Flavonoids	Flavonols
44	C27H30O16	Luteolin-7-O-gentiobioside	Flavonoids	Flavonoid
45	C30H26O12	Procyanidin B3*	Tannins	Proanthocyanidins
46	C30H26O12	Procyanidin B2*	Tannins	Proanthocyanidins
47	C16H12O5	izalpinin	Flavonoids	Flavonols
48	C16H14O5	7-O-Methylnaringenin	Flavonoids	Flavonoid
49	C9H8O3	α-Hydroxycinnamic Acid*	Phenolic acids	Phenolic acids
50	C14H20O9	Koaburaside	Phenolic acids	Phenolic acids
51	C28H20O14	Epitheaflavic acid-3-O-Gallate	Tannins	Tannin
52	C22H28O7	5-Hydroxy-1-(4-hydroxy-3,5-dimethoxyphenyl)-7-(4-hydroxy-3-metl	Others	Others
53	C16H12O7	Nepetin (5,7,3',4'-Tetrahydroxy-6-methoxyflavone)	Flavonoids	Flavonoid
54	C16H18O9	Chlorogenic acid*	Phenolic acids	Phenolic acids
55	C21H22O5	1,7-Bis(4-Hydroxy-3-Methoxyphenyl)Hepta-4,6-Dien-3-One	Phenolic acids	Phenolic acids
56	C28H34O12	Pinoresinol-4-O-(6"-acetyl)glucoside	ignans and Coumarin	Lignans
57	C24H24O13	Isorhamnetin-3-O-(6"-acetylglucoside)	Flavonoids	Flavonols
58	C28H32O16	2'-Hydoxy,5-methoxyGenistein-O-rhamnosyl-glucoside*	Flavonoids	Isoflavones
59	C27H30O15	Kaempferol-3-O-neohesperidoside*	Flavonoids	Flavonols
60	C31H29O14+	Petunidin-3-O-(6"-O-p-Coumaroyl)glucoside	Flavonoids	Anthocyanins
61	C16H12O7	Quercetin-3-O-methyl ether	Flavonoids	Flavonols

62	C14H14O8	Feruloylmalic acid	Phenolic acids	Phenolic acids
63	C28H32O16	Sexangularetin-3-O-glucoside-7-O-rhamnoside*	Flavonoids	Flavonols
64	C30H26O12	Procyanidin B4*	Tannins	Proanthocyanidins
65	C8H8O3	Vanillin	Phenolic acids	Phenolic acids
66	C15H20O10	Glucosyringic Acid	Phenolic acids	Phenolic acids
67	C17H22O10	1-O-Sinapoyl-D-glucose*	Phenolic acids	Phenolic acids
68	C18H16O6	5-Hydroxy-3,7,4'-trimethoxyflavone	Flavonoids	Flavonoid
69	C30H32O18	Luteolin-7-O-(6''-malonyl)glucoside-5-O-rhamnoside	Flavonoids	Flavonoid
70	C20H18O11	Quercetin-3-O-xyloside (Reynoutrin)*	Flavonoids	Flavonols
71	C16H12O6	Rhamnocitrin (7-Methylkaempferol)*	Flavonoids	Flavonoid
72	C21H22O6	Dihydrocurcumin	Others	Others
73	C8H7NO2	4-Hydroxymandelonitrile	Alkaloids	Alkaloids
74	C13H12O7	p-Coumaroylmalic acid	Phenolic acids	Phenolic acids
75	C30H26O14	Gallocatechin-Gallocatechin	Flavonoids	Flavanols
76	C5H11NO2	Betaine*	Alkaloids	Alkaloids
77	C20H18O11	Avicularin*	Flavonoids	Flavonols
78	C22H22O11	Diosmetin-7-O-galactoside	Flavonoids	Flavonoid
79	C23H24O12	Aurantio-obtusin-6-O-Glucoside	Quinones	Anthraquinone
80	C16H22O8	Coniferin	Phenolic acids	Phenolic acids
81	C8H9N	N-Benzylmethylene isomethylamine	Alkaloids	Alkaloids
82	C21H21O12+	Delphinidin-3-O-glucoside (Mirtillin)	Flavonoids	Anthocyanins
83	C16H20O10	Trihydroxycinnamoylquinic acid	Phenolic acids	Phenolic acids
84	C15H8O7	6-Hydroxyrhein	Quinones	Anthraquinone
85	C17H14O7	4',5,7-Trihydroxy-3',6-dimethoxyflavone (Jaceosidin)	Flavonoids	Flavonoid
86	C15H18O9	6-O-Caffeoyl-D-glucose*	Phenolic acids	Phenolic acids
87	C15H18O9	1-O-[(E)-Caffeoyl]-D-glucose*	Phenolic acids	Phenolic acids
88	C21H18O12	Scutellarein-7-O-glucuronide (Scutellarin)*	Flavonoids	Flavonoid
89	C21H18O12	Luteolin-7-O-glucuronide*	Flavonoids	Flavonoid
90	C15H14O6	Epicatechin*	Flavonoids	Flavanols
91	C9H8O3	p-Coumaric acid*	Phenolic acids	Phenolic acids
92	C14H18O9	1-O-Vanilloyl-D-Glucose	Phenolic acids	Phenolic acids
93	C21H20O11	Kaempferol-7-O-glucoside*	Flavonoids	Flavonols

94	C15H14O5	Phloretin	Flavonoids	Chalcones
95	C16H12O4	Tectochrysin	Flavonoids	Flavonoid
96	C7H6O3	Protocatechuic aldehyde	Phenolic acids	Phenolic acids
97	C18H28O9	5'-Glucosyloxyjasmanic acid	Phenolic acids	Phenolic acids
98	C20H22O3	yakuchinone B	Others	Others
99	C27H30O17	6-Hydroxykaempferol-7,6-O-Diglucoside	Flavonoids	Flavonols
100	C21H18O13	Quercetin-5-O-glucuronide*	Flavonoids	Flavonols
101	C15H10O4	Chrysin	Flavonoids	Flavonoid
102	C27H30O14	Kaempferol-3,7-O-dirhamnoside (Kaempferitrin)	Flavonoids	Flavonols
103	C20H24O4	5-hydroxy-1-phenyl-7- (4-hydroxy-3-methoxyphenyl) -3-heptanone	Others	Others
104	C22H28O7	5-Hydroxy-7-(4-hydroxy-3,5-dimethoxyphenyl)-1-(4-hydroxy-3-met[illegible]	Others	Others
105	C15H18O8	1-O-[(E)-p-Cumaroyl]-D-glucose*	Phenolic acids	Phenolic acids
106	C14H18O10	1-O-(3,4-Dihydroxy-5-methoxy-benzoyl)-glucoside	Phenolic acids	Phenolic acids
107	C16H14O4	Pinostrobin Chalcone*	Flavonoids	Chalcones
108	C23H20O14	Quercetin-3-O-(2''-acetyl)glucuronide	Flavonoids	Flavonols
109	C7H6O4	3,4-Dihydroxybenzoic acid (Protocatechuic acid)*	Phenolic acids	Phenolic acids
110	C6H6O3	5-Hydroxymethylfurfural	Phenolic acids	Phenolic acids
111	C8H9NO	N-benzylformamide	Alkaloids	Alkaloids
112	C14H22O	2,6-Di-t-butylphenol	Phenolic acids	Phenolic acids
113	C16H14O4	Isomethacin*	Alkaloids	Alkaloids
114	C21H24O11	Epicatechin glucoside	Flavonoids	Flavanols
115	C19H32O16	Di-O-Glucosylquinic acid	Phenolic acids	Phenolic acids
116	C16H22O9	Sweroside	Terpenoids	Sesquiterpenoids
117	C45H38O18	Arecatannin B1	Tannins	Tannin
118	C7H6O4	2,6-Dihydroxybenzoic acid*	Phenolic acids	Phenolic acids
119	C13H18O8	4-O-Glucosyl-3,4-dihydroxybenzyl alcohol	Phenolic acids	Phenolic acids
120	C13H16O8	4-O-Glucosyl-4-hydroxybenzoic acid	Phenolic acids	Phenolic acids
121	C26H28O16	Quercetin-3-O-sambubioside*	Flavonoids	Flavonols
122	C17H14O6	Kumatakenin	Flavonoids	Flavonols
123	C8H7NO3	2-(Formylamino)benzoic acid	Phenolic acids	Phenolic acids
124	C9H8O4	Caffeic acid	Phenolic acids	Phenolic acids
125	C14H20O8	5-(2-Hydroxyethyl)-2-O-glucosylphenol	Phenolic acids	Phenolic acids

126	$C_{28}H_{32}O_{16}$	Isorhamnetin-3-O-neohesperidoside*	Flavonoids	Flavonols
127	$C_{30}H_{26}O_{12}$	Procyanidin B1	Tannins	Proanthocyanidins
128	$C_{15}H_{12}O_{5}$	Naringenin (5,7,4'-Trihydroxyflavanone)*	Flavonoids	Dihydroflavone
129	$C_{11}H_{12}O_{4}$	Sinapinaldehyde	Phenolic acids	Phenolic acids
130	$C_{15}H_{12}O_{5}$	Pinobanksin*	Flavonoids	Dihydroflavonol
131	$C_{20}H_{24}O_{5}$	5-Hydroxy-1-(4-hydroxy-3-methoxyphenyl)-7-(4-hydroxyphenyl)hep	Others	Others
132	$C_{26}H_{28}O_{16}$	Quercetin-3-O-xylosyl(1→2)glucoside*	Flavonoids	Flavonols
133	$C_{23}H_{24}O_{11}$	5,2'-Dihydroxy-7,8-dimethoxyflavone glycosides	Flavonoids	Flavonoid
134	$C_{37}H_{39}O_{18}^{+}$	Petunidin-3-O-(6"-O-p-coumaroyl)glucoside-5-O-rhamnoside	Flavonoids	Anthocyanins
135	$C_{22}H_{22}O_{11}$	Chrysoeriol-5-O-glucoside	Flavonoids	Flavonoid
136	$C_{34}H_{42}O_{20}$	Rhamnetin-3-O-Rutinoside-5-O-rhamnoside*	Flavonoids	Flavonoid
137	$C_{34}H_{42}O_{20}$	Isorhamnetin-3-O-rutinoside-7-O-rhamnoside*	Flavonoids	Flavonoid
138	$C_{16}H_{18}O_{8}$	3-O-p-Coumaroylquinic acid*	Phenolic acids	Phenolic acids
139	$C_{33}H_{41}O_{21}^{+}$	Cyanidin-3-O-sophoroside-5-O-glucoside	Flavonoids	Anthocyanins
140	$C_{27}H_{30}O_{15}$	Kaempferol-3-O-rutinoside(Nicotiflorin)*	Flavonoids	Flavonoid
141	$C_{18}H_{16}O_{7}$	7,8-Dihydroxy-5,6,4'-trimethoxyflavone	Flavonoids	Flavonoid
142	$C_{7}H_{6}O_{2}$	4-Hydroxybenzaldehyde	Phenolic acids	Phenolic acids
143	$C_{21}H_{28}O_{6}$	1,7-Bis(4-hydroxy-3-methoxyphenyl)heptane-3,5-diol	Others	Others
144	$C_{15}H_{18}O_{8}$	p-Coumaric acid-4-O-glucoside*	Phenolic acids	Phenolic acids
145	$C_{29}H_{24}O_{12}$	Theaflavin	Tannins	Proanthocyanidins
146	$C_{21}H_{20}O_{11}$	Quercetin-3-O-rhamnoside(Quercitrin)*	Flavonoids	Flavonols
147	$C_{7}H_{6}O_{4}$	2,5-Dihydroxybenzoic acid*	Phenolic acids	Phenolic acids
148	$C_{15}H_{12}O_{7}$	Dihydroquercetin(Taxifolin)	Flavonoids	Dihydroflavonol
149	$C_{16}H_{20}O_{9}$	1-O-Feruloyl-D-Glucose*	Phenolic acids	Phenolic acids
150	$C_{31}H_{29}O_{14}^{+}$	Cyanidin-3-O-(6"-O-feruloyl)glucoside	Flavonoids	Anthocyanins
151	$C_{13}H_{16}O_{10}$	1-O-Galloyl-D-glucose	Phenolic acids	Phenolic acids
152	$C_{23}H_{20}O_{13}$	Kaempferol-3-O-(2"-O-acetyl)glucuronide	Flavonoids	Flavonols
153	$C_{15}H_{14}O_{6}$	Catechin*	Flavonoids	Flavanols
154	$C_{8}H_{4}O_{3}$	Phthalic anhydride	Phenolic acids	Phenolic acids
155	$C_{15}H_{12}O_{9}$	p-Dimeric galloyl methyl ester	Phenolic acids	Phenolic acids
156	$C_{25}H_{24}O_{14}$	Chrysoeriol-7-O-(6"-malonyl)glucoside*	Flavonoids	Flavonoid
157	$C_{8}H_{9}NO_{3}$	2-Amino-3-methoxybenzoic acid*	Phenolic acids	Phenolic acids

158	C16H18O8	5-O-p-Coumaroylquinic acid*	Phenolic acids	Phenolic acids
159	C16H14O6	Dihydrokaempferide	Flavonoids	Flavonols
160	C30H26O11	Epicatechin-epiafzelechin	Flavonoids	Flavanols
161	C20H22O6	Pinoresinol*	ignans and Coumari	Lignans
162	C10H10O4	Ferulic acid	Phenolic acids	Phenolic acids
163	C27H30O15	Luteolin-7-O-neohesperidoside (Lonicerin)*	Flavonoids	Flavonoid
164	C15H10O6	Kaempferol (3,5,7,4'-Tetrahydroxyflavone)	Flavonoids	Flavonols
165	C16H20O9	6-O-Feruloyl-D-glucose*	Phenolic acids	Phenolic acids
166	C30H24O12	2α,3α-Epoxy-5,7,3',4'-tetrahydroxyflavan-(4β-8-catechin)	Tannins	Proanthocyanidins
167	C20H30O3	Methyl-[8]-Shogaol	Phenolic acids	Phenolic acids
168	C20H22O4	1- (4-hydroxy-3-methoxyphenyl) -7-phenyl-3,5-diheptanone	Others	Others
169	C20H22O6	Epipinoresinol*	ignans and Coumari	Lignans
170	C8H6O4	Terephthalic acid*	Phenolic acids	Phenolic acids
171	C23H26O11	Persicoside	Flavonoids	Dihydroflavone
172	C16H16O6	3'-O-Methyl-(-)-epicatechin	Flavonoids	Flavanols
173	C7H8O2	4-Methylcatechol	Phenolic acids	Phenolic acids
174	C16H12O6	Hispidulin (5,7,4'-Trihydroxy-6-methoxyflavone)*	Flavonoids	Flavonoid
175	C16H18O9	Neochlorogenic acid(5-O-Caffeoylquinic acid)*	Phenolic acids	Phenolic acids
176	C21H44NO7P	3-{(2-Aminoethoxy)(hydroxy)phosphoryl]oxy}-2-hydroxypropyl pal	Alkaloids	Alkaloids
177	C15H12O5	1,7-diphenyl-2-hydroxy1-heptene	Others	Others
178	C28H30O17	Isorhamnetin-3-O-glucuronide-7-O-rhamnoside	Flavonoids	Flavonols
179	C17H20O11	Sinapoylglucuronic acid	Phenolic acids	Phenolic acids
180	C18H16O5	5,7,4'-Trimethoxyflavone	Flavonoids	Flavonoid
181	C9H10O4	Syringaldehyde; 4-Hydroxy-3,5-Dimethoxybenzaldehyde	Phenolic acids	Phenolic acids
182	C9H10O5	4-Hydroxy-3-methoxymandelate	Phenolic acids	Phenolic acids
183	C15H14O5	Epiafzelechin*	Flavonoids	Flavanols
184	C30H32O19	Kaempferol-3-O-(6"-Malonyl)glucoside-7-O-Glucoside	Flavonoids	Flavonols
185	C31H61O14N	3-Hydroxypropyl palmitate glc-glucosamine	Alkaloids	Alkaloids
186	C15H14O7	Epigallocatechin*	Flavonoids	Flavanols
187	C10H10O3	p-Coumaric acid methyl ester*	Phenolic acids	Phenolic acids
188	C15H10O7	Quercetin	Flavonoids	Flavonols
189	C15H12O6	Dihydrokaempferol	Flavonoids	Dihydroflavonol

190	C21H20O12	6-Hydroxykaempferol-7-O-glucoside*	Flavonoids	Flavonols
191	C8H8O4	Vanillic acid	Phenolic acids	Phenolic acids
192	C21H34O3	[10]-Paradol	Phenolic acids	Phenolic acids
193	C26H32O14	Phloretin-2'-O-(6"-O-xylosyl)glucoside	Flavonoids	Flavonoid
194	C28H42O8	Methyl-diacetoxy-[8]-gingerdiol	Phenolic acids	Phenolic acids
195	C15H10O7	Robinetin	Flavonoids	Flavonoid
196	C28H34O15	Hesperetin-7-O-rutinoside (Hesperidin)*	Flavonoids	Dihydroflavone
197	C28H32O17	Isorhamnetin-3,7-O-diglucoside	Flavonoids	Flavonols
198	C16H22O3	Methyl-[4]-Shogaol	Phenolic acids	Phenolic acids
199	C19H18O8	Feruloyl syringic acid	Phenolic acids	Phenolic acids
200	C19H24O4	1,7-Diphenylheptane-1,3,5,7-tetraol	Others	Others
201	C22H26O6	Gingerenone B	Others	Others
202	C8H7N	Indole	Alkaloids	Plumerane
203	C20H20O5	Dihydrodemethoxy curcumin	Others	Others
204	C26H32O12	Nortrachelogenin-4-O-glucoside	ignans and Coumarin	Lignans
205	C19H27NO12	Anthranilate-1-O-Sophoroside	Phenolic acids	Phenolic acids
206	C17H16O6	Cajanol	Flavonoids	Isoflavones
207	C20H39NO2	N-Oleoylethanolamine	Alkaloids	Alkaloids
208	C26H32O12	1-Hydroxypinoresinol-1-O-Glucoside	ignans and Coumarin	Lignans
209	C19H18O4	Dihydrodidemethoxy curcumin	Others	Others
210	C28H34O15	Hesperetin-7-O-neohesperidoside(Neohesperidin)*	Flavonoids	Dihydroflavone
211	C25H24O14	Diosmetin-7-O-(6"-malonyl)glucoside*	Flavonoids	Flavonoid
212	C30H28O12	Gambiriin A1	Tannins	Tannin
213	C9H16O4	Eucommiol*	Others	Others
214	C9H8O3	Caffeic aldehyde	Phenolic acids	Phenolic acids
215	C8H10O2	Tyrosol	Phenolic acids	Phenolic acids
216	C19H20O2	1,7-diphenyl-3,5-diheptanone	Others	Others
217	C19H16O3	1,7-Bis-(4-hydroxyphenyl)-2,4,6-heptatrienone	Phenolic acids	Phenolic acids
218	C13H20O3	Annuionone D	Others	Others
219	C10H10O2	4-MethoxycinnaMaldehyde	Phenolic acids	Phenolic acids
220	C10H10O3	Coniferaldehyde	Phenolic acids	Phenolic acids
221	C23H32O15	Furanofructosyl-α-D-(3-mustard acyl)glucoside	Phenolic acids	Phenolic acids

222	C5H5N5	2-Aminopurine	Alkaloids	Alkaloids
223	C12H18O3	Oxyphyllenone B	Terpenoids	Sesquiterpenoids
224	C16H18O9	Cryptochlorogenic acid*	Phenolic acids	Phenolic acids
225	C8H8O	4-Methylbenzaldehyde*	Others	Others
226	C10H14O2	1-(4-Methoxyphenyl)-1-propanol	Phenolic acids	Phenolic acids
227	C9H10O5	Syringic acid	Phenolic acids	Phenolic acids
228	C20H18O5	Demethoxycurcumin	Others	Others
229	C6H9NOS	4-Methyl-5-thiazoleethanol	Others	Others
230	C45H36O17	Epicatechin-(2β→O→7,4β→8)-epiafzelechin-(4α→8)-epicatechin	Flavonoids	Flavanols
231	C21H18O13	3,4-Digalloylshikimic acid*	Phenolic acids	Phenolic acids
232	C11H12O5	Sinapic acid	Phenolic acids	Phenolic acids
233	C18H24O12	Licoagroside B	Others	Others
234	C10H10O3	4-Methoxycinnamic acid*	Phenolic acids	Phenolic acids
235	C10H12O3	Coniferyl alcohol	Phenolic acids	Phenolic acids
236	C30H26O11	Gambiriin B3	Tannins	Tannin
237	C19H28O6	[4]-Gingerdiol	Phenolic acids	Phenolic acids
238	C10H10O4	Methyl caffeate	Phenolic acids	Phenolic acids
239	C17H27N3O17P2	Uridine 5'-diphospho-N-acetylglucosamine	Alkaloids	Alkaloids
240	C15H10O5	Apigenin	Flavonoids	Flavonoid
241	C33H40O19	Luteolin-7-O-(2"-O-rhamnosyl)rutinoside*	Flavonoids	Flavonoid
242	C17H22O9	Sinapaldehyde-4-O-Glucoside	Phenolic acids	Phenolic acids
243	C21H20O9	Chrysin-7-O-glucoside	Flavonoids	Flavonoid
244	C33H40O19	Kaempferol-3-O-robinoside-7-O-rhamnoside (Robinin)*	Flavonoids	Flavonols
245	C19H20O2	1-phenyl-7- (4-hydroxyphenyl) -4-ene-3-heptanone	Others	Others
246	C32H38O17	3,6'-Diferuloylsucrose	Phenolic acids	Phenolic acids
247	C19H22O3	5-hydroxy-1-phenyl-7- (4-hydroxyphenyl) -3-heptanone	Others	Others
248	C17H26O4	[6]-Gingerol	Phenolic acids	Phenolic acids
249	C15H10O5	Galangin	Others	Others
250	C20H26O3	oxyphyllacinol	Others	Others
251	C25H28O13	Syringic acid-4-O-(6"-feruloyl)glucoside	Phenolic acids	Phenolic acids
252	C9H10O2	Hydrocinnamic acid	Phenolic acids	Phenolic acids
253	C31H36O18	Syringoylcaffeoylquinic acid-D-glucose	Phenolic acids	Phenolic acids

254	C22H30O14	6'-O-Feruloyl-D-sucrose	Phenolic acids	Phenolic acids
255	C9H10O4	Methyl 2,4-dihydroxyphenylacetate	Phenolic acids	Phenolic acids
256	C8H8O	3-Methylbenzaldehyde*	Others	Others
257	C21H22O8	Nobiletin (5,6,7,8,3',4'-Hexamethoxyflavone)	Flavonoids	Flavonoid
258	C19H30O4	8-Gingerol	Others	Others
259	C15H16O9	Sinapoyl malate	Phenolic acids	Phenolic acids
260	C21H20O6	Curcumin	Others	Others
261	C17H24O4	[6]-Gingerdione	Phenolic acids	Phenolic acids
262	C16H12O7	Tamarixetin (3,3',5,7-Tetrahydroxy-4'-Methoxyflavone)	Flavonoids	Flavonoid
263	C22H26O12	5-O-p-Coumaroylshikimic acid O-glucoside	Phenolic acids	Phenolic acids
264	C9H8O2	Cinnamic acid	Phenolic acids	Phenolic acids
265	C21H20O10	Apigenin-8-C-Glucoside (Vitexin)	Flavonoids	Flavonoid carbonoside
266	C20H22O4	1- (4-hydroxyphenyl) -7- (4-hydroxy-3-methoxyphenyl) -4-ene-3-hep	Others	Others
267	C15H14O5	Afzelechin (3,5,7,4'-Tetrahydroxyflavan)*	Flavonoids	Flavanols
268	C20H20O7	Tangeretin	Flavonoids	Flavonols
269	C20H20O12	2-O-Salicyl-6-O-Galloyl-D-Glucose	Phenolic acids	Phenolic acids
270	C15H14O7	Gallocatechin*	Flavonoids	Flavanols
271	C7H6O2	Benzoic acid	Phenolic acids	Phenolic acids
272	C27H38O19	O-Caffeoyl maltotriose	Phenolic acids	Phenolic acids
273	C19H38N2O3	Cocamidopropyl betaine	Alkaloids	Alkaloids
274	C20H24O3	yakuchinone A	Others	Others
275	C30H46O5	Isoceanothic acid	Terpenoids	Triterpene
276	C20H24O6	(+)-Isolariciresinol	ignans and Coumarin	Lignans
277	C15H22O3	oxyphyllol E	Terpenoids	Sesquiterpenoids
278	C15H22O9	Aucubin	Terpenoids	Sesquiterpenoids
279	C22H22O8	1-O-Feruloyl-3-O-p-Coumaroylglycerol	Phenolic acids	Phenolic acids
280	C19H20O	1,7-diphenyl-4-ene-3-heptanone	Others	Others
281	C9H10O3	3-(4-Hydroxyphenyl)-propionic acid	Phenolic acids	Phenolic acids
282	C10H8O3	6-Hydroxy-4-methylcoumarin	ignans and Coumarin	Coumarins
283	C11H12O2	Ethyl cinnamate	Phenolic acids	Phenolic acids
284	C13H18O2	4-Hydroxy-3,5-diisopropylbenzaldehyde	Phenolic acids	Phenolic acids
285	C11H12O4	Ethyl caffeate*	Phenolic acids	Phenolic acids

286	C11H12O4	3,4-Dimethoxycinnamic acid*	Phenolic acids	Phenolic acids
287	C12H18O3	oxyphyllenone A	Terpenoids	Sesquiterpenoids
288	C14H18O2	oxyphyllone D	Terpenoids	Sesquiterpenoids
289	C14H18O2	oxyphyllone G	Terpenoids	Sesquiterpenoids
290	C15H24O	nootkatol	Terpenoids	Sesquiterpenoids
291	C15H24O	epinootkatol	Terpenoids	Sesquiterpenoids
292	C15H24O	oxyphyllol A	Terpenoids	Sesquiterpenoids
293	C14H20O3	Oxyphyllenotriol A	Terpenoids	Sesquiterpenoids
294	C11H10O6	Benzoylmalic acid	Phenolic acids	Phenolic acids
295	C14H22O3	oxyphyllenodiol A	Terpenoids	Sesquiterpenoids
296	C15H26O2	Oxyphyllol C	Terpenoids	Sesquiterpenoids
297	C15H18O3	Hydroxyindesteolide	Others	Others
298	C15H24O3	(11S)-NOOTKATONE-11,12-DIOL	Terpenoids	Sesquiterpenoids
299	C11H10O7	p-Hydroxybenzoylmalic acid	Phenolic acids	Phenolic acids
300	C19H18O	1,7-diphenyl-4,6-diene-3-heptanone	Others	Others
301	C14H20NO4+	Caffeoylcholine	Alkaloids	Alkaloids
302	C17H26O3	[6]-Paradol	Phenolic acids	Phenolic acids
303	C16H24O4	[5]-Gingerol	Phenolic acids	Phenolic acids
304	C19H22O2	5-hydroxy-1,7-diphenyl-3-heptanone	Others	Others
305	C12H12O8	Vnilloylmalic acid	Phenolic acids	Phenolic acids
306	C19H28O3	[8]-Shogaol	Phenolic acids	Phenolic acids
307	C14H14O9	3-Galloylshikimic acid	Phenolic acids	Phenolic acids
308	C21H32O3	[10]-Shogaol	Phenolic acids	Phenolic acids
309	C22H37NO2	N-(2-hydroxyethyl)-5,8,11,14-eicosatrienoic amide	Others	Others
310	C17H24O9	Syringin	Phenolic acids	Phenolic acids
311	C24H22O12	p-Coumaroylsinapoyltartaric acid	Phenolic acids	Phenolic acids
312	C28H32O14	Acacetin-7-O-rutinoside (Linarin)	Flavonoids	Flavonoid
