# Supplementary material for: Analysis of Bioactive Components in the Fruit, Roots, and Leaves of Alpinia oxyphylla by UPLC-MS/MS
Source: Evid Based Complement Alternat Med. 2021 Jul 9;2021:5592518. doi: 10.1155/2021/5592518 (PMC8286198; doi:10.1155/2021/5592518)
Supplement: Supplementary Materials — Figure S1: MRM metabolite detection. The multipeak diagram shows the substances that were detected in the sample, and each mass spectral peak with different colors represents one detected metabolite. Figure S2: OPLS-DA analysis model verification diagram. Table S1: 312 identified metabolites. Table S2: the target genes were enriched in multiple pathways. [file 5592518.f1.zip › 5592518.f1/Table S2.pdf]

Table S2 The target gene were enriched in multiple pathways								
	**Biological process**	**No. of genes in the dataset**	**No. of genes in the background dataset**	**Percentage of genes**	**Fold enrichment**	**P-value (Hypergeometric test)**	**Bonferroni method**	**genes mapped (from input data set)**
Fruit	Metabolism	130	1683	42.4837	4.57519	1.6E-54	2.8E-52	XDH; TYR; SQLE; SI; QDPR; PYGM; PYGB; PON1; PNLIP; PLCG1; PLA2G1B; PGD; PDK3; PARG; ODC1; NUAK1; NQO1; NOX4; NOS3; NDUFV3; NDUFV2; NDUFV1; NDUFS8; NDUFS7; NDUFS6; NDUFS5; NDUFS4; NDUFS3; NDUFS2; NDUFS1; NDUFC2; NDUFC1; NDUFB9; NDUFB8; NDUFB7; NDUFB6; NDUFB5; NDUFB4; NDUFB3; NDUFB2; NDUFB11; NDUFB10; NDUFB1; NDUFAB1; NDUFA9; NDUFA8; NDUFA6; NDUFA5; NDUFA4L2; NDUFA4; NDUFA3; NDUFA2; NDUFA13; NDUFA12; NDUFA11; NDUFA10; NDUFA1; MT-ND5; MPO; MMEL1; MGAM; MDH2; MDH1; MAOB; MAOA; LPO; LCT; HSD17B3; HSD17B2; HSD17B1; HSD11B1; HK2; HK1; HEXB; HEXA; GUSB; GSK3B; GLO1; GLB1; GLA; GANC; GAA; FUT7; FUT4; FUCA1; FKBP4; FASN; FADS1; CYP2C8; CYP2A6; CYP27B1; CYP24A1; CYP1B1; CYP1A1; CDA; CD38; CBR1; CA9; CA7; CA6; CA5B; CA5A; CA4; CA3; CA2; CA14; CA13;
	Energy pathways	128	1633	41.8301	4.64273	2.8E-54	5E-52	XDH; TYR; SQLE; SI; QDPR; PYGM; PYGB; PON1; PNLIP; PLCG1; PLA2G1B; PGD; PDK3; PARG; ODC1; NUAK1; NQO1; NOX4; NOS3; NDUFV3; NDUFV2; NDUFV1; NDUFS8; NDUFS7; NDUFS6; NDUFS5; NDUFS4; NDUFS3; NDUFS2; NDUFS1; NDUFC2; NDUFC1; NDUFB9; NDUFB8; NDUFB7; NDUFB6; NDUFB5; NDUFB4; NDUFB3; NDUFB2; NDUFB11; NDUFB10; NDUFB1; NDUFAB1; NDUFA9; NDUFA8; NDUFA6; NDUFA5; NDUFA4L2; NDUFA4; NDUFA3; NDUFA2; NDUFA13; NDUFA12; NDUFA11; NDUFA10; NDUFA1; MT-ND5; MPO; MMEL1; MGAM; MDH2; MDH1; MAOB; MAOA; LPO; LCT; HSD17B3; HSD17B2; HSD17B1; HK2; HK1; HEXB; HEXA; GUSB; GSK3B; GLO1; GLB1; GLA; GANC; GAA; FUT7; FUT4; FUCA1; FKBP4; FASN; FADS1; CYP2C8; CYP2A6; CYP27B1; CYP24A1; CYP1B1; CYP1A1; CDA; CD38; CBR1; CA9; CA6; CA5B; CA5A; CA4; CA3; CA2; CA14; CA13; CA12; BCHE;
	Carbohydrate metabolism	2	8	0.65359	14.8622	0.00744	1	PDK4; AGL;
	Lymphocyte activation	1	1	0.3268	59.2269	0.01688	1	ST6GAL1;

	Lymphocyte proliferation	1	1	0.3268	59.2269	0.01688	1	ST6GAL1;
	Cell communication	78	3713	25.4902	1.24435	0.01905	1	VEGFA; TRPV1; TNKS2; TNFRSF1A; TNF; TLR2; TLR1; TAS2R31; STK17B; ST3GAL3; PTPRS; PTPN7; PTGFR; PRKDC; PLAA; PKN1; PGF; PDGFRA; OPRD1; NR3C2; NOD2; NMUR2; NEK6; NDUFAF4; NAE1; MYLK; MNAT1; MIF; MET; MAP3K7; LGALS4; ITPR1; INHA; IKBKG; IGFBP5; IGF2R; HTR2C; HTR2B; HTR2A; HRAS; HES1; HCN1; GRK6; GPR35; GFER; GCGR; FGF2; FGF1; FFAR4; ERN1; EGFR; EDNRB; EDNRA; DYRK4; DYRK1A; DUSP3; DRD1; DBH; DAPK1; CSNK2B; CSNK2A2; CCR4; CAMK2B; CAMK2A; CALM1; BRAF; AXL;
	Signal transduction	82	3934	26.7974	1.23467	0.01923	1	VEGFA; TRPV1; TNKS2; TNFRSF1A; TNF; TLR2; TLR1; TAS2R31; STK17B; ST3GAL3; SRC; PTPRS; PTPN7; PTGFR; PRKDC; PLAA; PKN1; PGF; PDGFRA; OPRD1; NR3C2; NOD2; NMUR2; NEK6; NDUFAF4; NAE1; MYLK; MNAT1; MIF; MET; MAP3K7; MAP2K6; LGALS8; LGALS4; ITPR1; INHA; IKBKG; IGFBP5; IGF2R; HTR2C; HTR2B; HTR2A; HRAS; HES1; HCN1; GRK6; GPR35; GFER; GCGR; FGF2; FGF1; FFAR4; ERN1; EGFR; EDNRB; EDNRA; DYRK4; DYRK1A; DUSP3; DRD1; DBH; DAPK1; CSNK2B; CSNK2A2; CLK1; CCR4; CAMK2B; CAMK2A; CALM1; BRAF; AXL; ARPP19; AR; APP; ADRB3; ADRB2;
	Inflammatory response	2	14	0.65359	8.49721	0.02262	1	TRPV1; LGALS9;
	Neurotransmitter metabolism	1	2	0.3268	29.7608	0.03348	1	ACHE;
	Vitamin metabolism	1	2	0.3268	29.7608	0.03348	1	VKORC1;
	Transport	29	1215	9.47712	1.41412	0.03776	1	TTR; TRPM8; TRPA1; SYN1; SLC8A1; SLC6A4; SLC6A3; SLC6A13; SLC6A11; SLC5A5; SLC5A4; SLC5A2; SLC5A11; SLC5A1; SLC37A4; SLC28A3; SLC22A3; SLC1A3; SLC16A7; SLC16A3; SHBG; NPC1L1; KCNK2; KCND3; HCN4; ABCG2;
	Hormone metabolism	1	3	0.3268	19.8735	0.0498	1	CYP19A1;

	Metabolism	144	1683	35.468	3.81965	9.5E-49	1.7E-46	PGD; ACHE; MAOA; MAOB; GUSB; CA3; CA4; CA7; CBR1; CYP1B1; CYP24A1; ODC1; HSD17B2; FADS1; FUT4; FUT7; PNLIP; ALOX12; TYR; NDUFAB1; AKR1B10; CA13; CA5A; CA6; CA5B; HSD17B3; QDPR; FKBP4; GSK3B; GLO1; ALOX15; ALOX15B; MMEL1; NDUFA1; NDUFA2; NDUFA3; NDUFA4; NDUFA5; NDUFA6; NDUFA8; NDUFA9; NDUFA10; NDUFA11; NDUFA13; NDUFA12; NDUFB1; NDUFB2; NDUFB3; NDUFB4; NDUFB5; NDUFB6; NDUFB7; NDUFB8; NDUFB10; NDUFB9; NDUFB11; NDUFC1; NDUFC2; NDUFS1; NDUFS2; NDUFS3; NDUFS4; NDUFS5; NDUFS6; NDUFS7; NDUFS8; NDUFV1; NDUFV2; NDUFV3; MT-ND5; NDUFA4L2; BCHE; ARG1; SQLE; NOS3; NDUFA7; AKR1B1; AKR1A1; ALDH2; AMY1A; CA12; CYP2C8; FASN; ALOX5; MDH1; MDH2; NOX4; NQO1; NUAK1; PDK3; LPO; MPO; PON1; ACP1; ALPI; XDH; AKR1C4; CA14; CYP27B1; HSD11B1; AKR1C3; CA9; CYP1A1; NOS2; SPR; ACER2; ASAH1; CEL; CYP1A2; CYP2C9; CYP2C19; CES1; CES2; FAAH; GSTM1; EPHX1; EPHX2; KYNU; NAAA; NR1H4;
	Energy pathways	137	1633	33.7438	3.74525	4.8E-45	8.6E-43	PGD; ACHE; MAOA; MAOB; GUSB; CA3; CA4; CBR1; CYP1B1; CYP24A1; ODC1; HSD17B2; FADS1; FUT4; FUT7; PNLIP; ALOX12; TYR; NDUFAB1; AKR1B10; CA13; CA5A; CA6; CA5B; HSD17B3; QDPR; FKBP4; GSK3B; GLO1; ALOX15; ALOX15B; MMEL1; NDUFA1; NDUFA2; NDUFA3; NDUFA4; NDUFA5; NDUFA6; NDUFA8; NDUFA9; NDUFA10; NDUFA11; NDUFA13; NDUFA12; NDUFB1; NDUFB2; NDUFB3; NDUFB4; NDUFB5; NDUFB6; NDUFB7; NDUFB8; NDUFB10; NDUFB9; NDUFB11; NDUFC1; NDUFC2; NDUFS1; NDUFS2; NDUFS3; NDUFS4; NDUFS5; NDUFS6; NDUFS7; NDUFS8; NDUFV1; NDUFV2; NDUFV3; MT-ND5; NDUFA4L2; BCHE; ARG1; SQLE; NOS3; NDUFA7; AKR1B1; ALDH2; AMY1A; CA12; CYP2C8; FASN; ALOX5; MDH1; MDH2; NOX4; NQO1; NUAK1; PDK3; LPO; MPO; PON1; ACP1; ALPI; XDH; AKR1C4; CA14; CYP27B1; AKR1C3; CA9; CYP1A1; NOS2; SPR; ACER2; ASAH1; CEL; CYP1A2; CYP2C9; CYP2C19; FAAH; GSTM1; EPHX1; EPHX2;

Leaf	Signal transduction	128	3934	31.5271	1.45253	2.1E-06	0.00038	ACVRL1; BRAF; DRD1; EDNRA; EDNRB; MAP3K7; MAP2K6; PDGFRA; PLAA; ST3GAL3; STK17B; TAS2R31; HTR2B; HTR2C; APP; ADORA2B; ADRB1; ADRB2; ADRB3; ARPP19; DUSP3; FFAR4; IGFBP5; NDUFAF4; IKBKG; NOD2; PTGFR; PTPN7; SRC; TRPV1; PGF; VEGFA; SELL; SELP; TLR1; TLR2; CALM1; DYRK4; CAMK2A; MET; DAPK1; DYRK1A; EGFR; ERN1; GPR35; GRK6; MYLK; NEK6; NMUR2; OPRD1; PKN1; PTPRS; HTR2A; GFER; GCGR; HCN1; INHA; IGF2R; TNFRSF1A; DNM1; ADCYAP1R1; PTPN1; PTPN6; TLR4; TLR9; HTR4; ADORA1; ADORA3; CHRM1; CHRM3; CHRM4; CHRM5; AGTR1; CACNA1D; CNR1; CNR2; DBH; GABRB2; GPR34; GRM2; GRM6; RASGRP1; HRH1; HRH2; HRH3; PRKCA; PRKCD; MIF; CDC25A; CDC25B; CDC25C; MTNR1A; MTNR1B; NR1H4; OXER1; PTGDR; PTGER1; PTGER2; PTAFR; NCOA3; RARA; RARB; S1PR4; S1PR5; SMO; TAOK3; THRB; TLR8; TSPO;
	Cell communication	118	3713	29.064	1.41876	2.1E-05	0.00366	ACVRL1; BRAF; DRD1; EDNRA; EDNRB; MAP3K7; PDGFRA; PLAA; ST3GAL3; STK17B; TAS2R31; HTR2B; HTR2C; APP; ADORA2B; ADRB1; ADRB2; ADRB3; ARPP19; DUSP3; FFAR4; IGFBP5; NDUFAF4; IKBKG; NOD2; PTGFR; PTPN7; TRPV1; PGF; VEGFA; SELL; SELP; TLR1; TLR2; CALM1; DYRK4; CAMK2A; MET; DAPK1; DYRK1A; EGFR; ERN1; GPR35; GRK6; MYLK; NEK6; NMUR2; OPRD1; PKN1; PTPRS; HTR2A; GFER; GCGR; HCN1; INHA; IGF2R; TNFRSF1A; DNM1; ADCYAP1R1; PTPN1; PTPN6; TLR4; HTR4; ADORA1; ADORA3; CHRM1; CHRM3; CHRM4; CHRM5; AGTR1; CACNA1D; CNR1; CNR2; DBH; GABRB2; GPR34; GRM2; GRM6; RASGRP1; HRH2; HRH3; PRKCA; PRKCD; MIF; CDC25A; CDC25B; CDC25C; MTNR1A; MTNR1B; NR1H4; OXER1; PTGDR; PTGER1; PTGER2; PTAFR; NCOA3; RARB; S1PR4; S1PR5; SMO; TAOK3; THRB; TLR8;
	Xenobiotic metabolism	3	6	0.73892	22.3568	0.00021	0.03778	NQO1; CYP2D6; GSTM1;
	Transport	40	1215	9.85222	1.46996	0.00942	1	ABCG2; SLC1A3; KCND3; ABCB1; SLC8A1; NPC1L1; SLC5A5; TRPA1; KCNK2; SLC22A3; SLC6A11; SLC6A13; SHBG; TRPM8; TTR; SLC6A4; SLC16A3; ABCC1; SLC16A7; SYN1; HCN4; SLC6A3; SLC5A2; TRPM5; CACNA1C; CACNA1S; CNGA1; GABRA6; GABRB1; GABRG2; KCNQ1; MTTP; SLC22A6; SLC6A5; SCN3A; SCN8A; SLC37A4; ABCC2; KCNB1; NOX1;
	Lymphocyte activation	1	1	0.24631	44.6393	0.0224	1	ST6GAL1;
	Lymphocyte proliferation	1	1	0.24631	44.6393	0.0224	1	ST6GAL1;
	DNA replication	2	13	0.49261	6.89662	0.03317	1	POLH; POLI;
	Lipid metabolism	3	33	0.73892	4.07041	0.03707	1	PTGS1; PTGES; MGLL;
	Neurotransmitter metabolism	1	2	0.24631	22.4307	0.0443	1	ACHE;

	Steroid hormone receptor signaling pathway	1	2	0.24631	22.4307	0.0443	1	NCOA1;
	Vitamin metabolism	1	2	0.24631	22.4307	0.0443	1	VKORC1;
	Respiratory electron transport	45	76	15.625	12.9325	9.7E-42	1.6E-38	NDUFA7; MT-ND1; NDUFAB1; NDUFA1; NDUFA2; NDUFA3; NDUFA4; NDUFA5; NDUFA6; NDUFA8; NDUFA9; NDUFA10; NDUFA11; NDUFA13; NDUFA12; NDUFB1; NDUFB2; NDUFB3; NDUFB4; NDUFB5; NDUFB6; NDUFB7; NDUFB8; NDUFB10; NDUFB9; NDUFB11; NDUFC1; NDUFC2; NDUFS1; NDUFS2; NDUFS3; NDUFS4; NDUFS5; NDUFS6; NDUFS7; NDUFS8; NDUFV1; NDUFV2; NDUFV3; MT-ND2; MT-ND3; MT-ND4L;
	Respiratory electron transport, ATP synthesis by chemiosmotic coupling, and heat production by uncoupling proteins.	45	91	15.625	10.801	5.8E-37	9.6E-34	NDUFA7; MT-ND1; NDUFAB1; NDUFA1; NDUFA2; NDUFA3; NDUFA4; NDUFA5; NDUFA6; NDUFA8; NDUFA9; NDUFA10; NDUFA11; NDUFA13; NDUFA12; NDUFB1; NDUFB2; NDUFB3; NDUFB4; NDUFB5; NDUFB6; NDUFB7; NDUFB8; NDUFB10; NDUFB9; NDUFB11; NDUFC1; NDUFC2; NDUFS1; NDUFS2; NDUFS3; NDUFS4; NDUFS5; NDUFS6; NDUFS7; NDUFS8; NDUFV1; NDUFV2; NDUFV3; MT-ND2; MT-ND3; MT-ND4L;
	The citric acid (TCA) cycle and respiratory electron transport	48	118	16.6667	8.88498	2.1E-34	3.5E-31	NDUFA7; MT-ND1; NDUFAB1; NDUFA1; NDUFA2; NDUFA3; NDUFA4; NDUFA5; NDUFA6; NDUFA8; NDUFA9; NDUFA10; NDUFA11; NDUFA13; NDUFA12; NDUFB1; NDUFB2; NDUFB3; NDUFB4; NDUFB5; NDUFB6; NDUFB7; NDUFB8; NDUFB10; NDUFB9; NDUFB11; NDUFC1; NDUFC2; NDUFS1; NDUFS2; NDUFS3; NDUFS4; NDUFS5; NDUFS6; NDUFS7; NDUFS8; NDUFV1; NDUFV2; NDUFV3; MT-ND2; MT-ND3; MT-ND4L; MT-ND4; MT-ND5; MT-ND6; MDH2; PDK4;
	Metabolism	84	823	29.1667	2.22931	1.3E-13	2.1E-10	MAOA; ARG1; CYP1B1; CYP2C8; ELAVL1; FASN; XDH; NT5E; QDPR; CYP2A6; NDUFA7; HAO1; HPD; PRKCA; MT-ND1; PAOX; SLC6A8; ADA; ADK; CDA; ALDH1A1; HPRT1; IDO1; NDUFAB1; FFAR4; NDUFA1; NDUFA2; NDUFA3; NDUFA4; NDUFA5; NDUFA6; NDUFA8; NDUFA9; NDUFA10; NDUFA11; NDUFA13; NDUFA12; NDUFB1; NDUFB2; NDUFB3; NDUFB4; NDUFB5; NDUFB6; NDUFB7; NDUFB8; NDUFB10; NDUFB9; NDUFB11; NDUFC1; NDUFC2; NDUFS1; NDUFS2; NDUFS3; NDUFS4; NDUFS5; NDUFS6; NDUFS7; NDUFS8; NDUFV1; NDUFV2; NDUFV3; MT-ND2; MT-ND3; MT-ND4L; MT-ND4; MT-ND5; MT-ND6; TPR; CYP19A1; ODC1; MAOB; CYP1A1;
	LPA receptor mediated events	25	100	8.68056	5.46153	1.3E-12	2.2E-09	IL6; HRAS; CREB1; MAPT; NFKB1; GNAI1; GNAI3; GNAO1; PRKCA; LPAR1; LPAR3; LPAR2; LPAR4; PLCG1; FOS; GSK3B; JUN; IKBKG; NOD2; SRC; MMP2; MMP9; TNFRSF1A; EGFR;
	Metabolism of steroid hormones and vitamins A and D	10	32	3.47222	6.82955	9.1E-07	0.00152	AKR1B1; HSD17B1; HSD17B3; PLB1; PNLIP; CYP19A1; CYP24A1; CYP27B1; HSD11B1; CYP17A1;

Nongenotropic Androgen signaling	9	26	3.125	7.56534	1.2E-06	0.00205	HRAS; CREB1; GNAI1; GNAI3; GNAO1; FOS; SHBG; SRC; AR;
ATF-2 transcription factor network	13	59	4.51389	4.81499	1.8E-06	0.003	ARG1; IL6; CREB1; ESR1; PRKCA; ACHE; DUSP1; EP300; FOS; JUN; HES1; MMP2; NOS2;
Phase 1 - Functionalization of compounds	13	60	4.51389	4.73475	2.2E-06	0.00367	MAOA; CYP1B1; CYP2C8; CYP2A6; PAOX; ALDH1A1; CYP19A1; MAOB; CYP1A1; CYP24A1; CYP27B1; CYP17A1;
Metabolism of carbohydrates	16	92	5.55556	3.80014	3.3E-06	0.00553	SLC5A1; AMY2A; AGL; HK1; HK2; LCT; MGAM; PYGB; PYGM; SI; TPR; PGD; MDH1; MDH2; SLC37A4; CALM1;
estrogen biosynthesis	4	4	1.38889	21.8396	4.3E-06	0.00719	HSD17B1; HSD17B3; CYP19A1; AKR1C3;
HIF-1-alpha transcription factor network	13	66	4.51389	4.30439	6.8E-06	0.01135	ABCG2; CA9; ABCB1; VEGFA; NT5E; CREB1; TERT; HK1; HK2; EP300; FOS; JUN; NOS2;
Steroid hormones	8	25	2.77778	6.9946	9.6E-06	0.016	AKR1B1; HSD17B1; HSD17B3; CYP19A1; CYP24A1; CYP27B1; HSD11B1; CYP17A1;
Glucocorticoid receptor regulatory network	14	80	4.86111	3.82417	1.3E-05	0.02128	IL2; IL5; IL6; CREB1; NFKB1; EP300; FKBP4; FOS; GSK3B; JUN; HDAC1; NR3C1; NR1I3; RELA;
Digestion of dietary	4	5	1.38889	17.4804	2.1E-05	0.03463	AMY2A; LCT; MGAM; SI;
phenylalanine degradation IV (mammalian, via side chain)	4	5	1.38889	17.4804	2.1E-05	0.03463	MAOA; HPD; MAOB; ALDH2;
Glucocorticoid receptor signaling	14	85	4.86111	3.59925	2.6E-05	0.0436	IL2; IL5; IL6; CREB1; NFKB1; EP300; FKBP4; FOS; GSK3B; JUN; HDAC1; NR3C1; NR1I3; RELA;
IL1-mediated signaling events	25	234	8.68056	2.33412	5.8E-05	0.09756	IL2; IL5; PKN1; CREB1; ESR1; CAMK2B; MAPT; NFKB1; PRKCA; PRKCH; CDC25B; PLA2G4A; DUSP1; EP300; FOS; GSK3B; JUN; IKBKG; NOD2; SRC; NOS2; PTPN1; TNFRSF1A;
Hypoxic and oxygen homeostasis regulation of HIF-1-	13	80	4.51389	3.55121	5.9E-05	0.09815	ABCG2; CA9; ABCB1; VEGFA; NT5E; CREB1; TERT; HK1; HK2; EP300; FOS; JUN; NOS2;
Neurotransmitter Clearance In The Synaptic Cleft	4	6	1.38889	14.5718	6E-05	0.10015	MAOA; SLC22A2; ACHE; ALDH2;
tryptophan degradation X (mammalian, via tryptamine)	4	6	1.38889	14.5718	6E-05	0.10015	AKR1A1; MAOA; MAOB; ALDH2;
Androgen-mediated signaling	17	130	5.90278	2.8574	8E-05	0.13266	PKN1; HRAS; CREB1; GNAI1; GNAI3; GNAO1; EP300; FKBP4; FOS; GSK3B; JUN; HDAC1; SHBG; SRC; KLK2; AR; NR3C1;
Transport of glucose and other sugars, bile salts and organic acids, metal ions and amine compounds	14	94	4.86111	3.25468	8.3E-05	0.13785	SLC16A7; SLC5A1; SLC5A2; SLC22A1; SLC22A2; SLC22A6; SLC22A8; SLC22A3; SLC6A11; SLC6A13; SLC16A3; SLC13A2; SLC13A3; SLC13A5;
Bile salt and organic anion SLC transporters	5	12	1.73611	9.11042	0.00012	0.1969	SLC16A7; SLC16A3; SLC13A2; SLC13A3; SLC13A5;
Organic cation/anion/zwitterion transport	5	12	1.73611	9.11042	0.00012	0.1969	SLC22A1; SLC22A2; SLC22A6; SLC22A8; SLC22A3;
serotonin degradation	4	7	1.38889	12.4931	0.00014	0.22525	MAOA; ADH1B; MAOB; ALDH2;
noradrenaline and adrenaline degradation	4	7	1.38889	12.4931	0.00014	0.22525	MAOA; ADH1B; MAOB; ALDH2;

CXCR4-mediated signaling events	21	190	7.29167	2.41487	0.00014	0.23885	IL2; HRAS; GRK6; NFKB1; DNM1; GNAI1; GNAI3; GNAO1; PRKCA; PLCG1; FOS; JUN; IKBKG; NOD2; PTPN7; SRC; MMP9; PTPN6; TNFRSF1A; RELA; CALM1;
IL23-mediated signaling events	11	66	3.81944	3.64268	0.00017	0.28854	IL2; IL6; MPO; NFKB1; PRKCA; IKBKG; NOD2; SRC; NOS2; TNFRSF1A; RELA;
Lysosphingolipid and LPA receptors	4	8	1.38889	10.9334	0.00026	0.43429	LPAR1; LPAR3; LPAR2; S1PR2;
AP-1 transcription factor network	47	621	16.3194	1.65324	0.00032	0.53119	ABCG2; ARG1; CA9; IL2; IL5; IL6; ABCB1; PKN1; HRAS; VEGFA; NT5E; CREB1; ESR1; CAMK2B; MAPT; TERT; NFKB1; PRKCA; PRKCH; CDC25B; PLA2G4A; HK1; HK2; ACHE; DUSP1; EP300; FKBP4; FOS; GSK3B; JUN; PTPN7; HDAC1; SRC; HES1; KLK2; CAMK2A; MMP2; MMP9; NOS2; PTPN1; AR; NR3C1; NR1I3; RBBP4; RBBP7; RELA; CALM1;
Adenosine P1 receptors	3	4	1.04167	16.3933	0.00037	0.61253	ADORA1; ADORA3; ADORA2B;
Amine Oxidase reactions	3	4	1.04167	16.3933	0.00037	0.61253	MAOA; PAOX; MAOB;
p38 MAPK signaling pathway	20	189	6.94444	2.3121	0.00037	0.61661	IL2; IL5; PKN1; CREB1; ESR1; CAMK2B; MAPT; PRKCA; PRKCH; CDC25B; PLA2G4A; DUSP1; EP300; FOS; GSK3B; JUN; SRC; NOS2; PTPN1; CALM1;
Transmission across Chemical Synapses	15	121	5.20833	2.70896	0.00038	0.63129	MAOA; HRAS; CREB1; CAMK2B; GNAI1; GNAI3; PLA2G4A; SLC22A2; ACHE; SLC6A11; SLC6A13; CAMK2A; GLUL; ALDH2; CALM1;
Fc-epsilon receptor I signaling in mast cells	10	61	3.47222	3.58325	0.00039	0.65329	HRAS; PLA2G1B; NFKB1; PLA2G4A; DUSP1; PLCG1; FOS; JUN; IKBKG; RELA;
Nucleotide-like (purinergic) receptors	5	15	1.73611	7.28955	0.0004	0.66628	ADORA1; LPAR4; LPAR6; ADORA3; ADORA2B;
N-cadherin signaling events	24	251	8.33333	2.08903	0.00045	0.75441	FGF1; HRAS; TERT; CNR1; DAGLA; PLCG1; EP300; FKBP4; FOS; GSK3B; JUN; HDAC1; SRC; KLK2; CAMK2A; MET; MMP2; MMP9; PTPN1; PTPN6; AR; EGFR; LRP6; CALM1;
Biological oxidations	13	98	4.51389	2.89902	0.00048	0.79816	MAOA; CYP1B1; CYP2C8; CYP2A6; PAOX; ALDH1A1; CYP19A1; MAOB; CYP1A1; CYP24A1; CYP27B1; CYP17A1;
TNF receptor signaling pathway	27	299	9.375	1.9728	0.0005	0.83508	IL2; IL5; PKN1; TOP1; CREB1; ESR1; CAMK2B; MAPT; NFKB1; PRKCA; PRKCH; CDC25B; PLA2G4A; DUSP1; APP; EP300; FOS; GSK3B; JUN; IKBKG; NOD2; SRC; NOS2; PTPN1; TNFRSF1A; RELA; CALM1;
Metabolism of lipids and lipoproteins	24	257	8.33333	2.04026	0.00064	1	AKR1B1; FASN; ALOX5; ABCC1; P4HB; HSD17B1; ACER2; HSD17B3; LSS; HMGCR; SLC25A20; MGLL; PLB1; PNLIP; NPC1L1; CYP19A1; AKR1C4; CYP1A1; CYP24A1; CYP27B1; HSD11B1; CYP17A1; DHCR7; EBP;
TCR signaling in naïve CD8+ T cells	15	127	5.20833	2.58099	0.00064	1	IL2; HRAS; NFKB1; PRKCA; PLCG1; FOS; JUN; IKBKG; NOD2; PTPN7; SRC; PTPN6; TNFRSF1A; RELA; CALM1;
superpathway of cholesterol biosynthesis	6	25	2.08333	5.24813	0.00074	1	LSS; FDPS; GGPS1; HMGCR; DHCR7; EBP;

IL2 signaling events mediated by PI3K	10	67	3.47222	3.26241	0.00084	1	IL2; TERT; NFKB1; PRKCA; IKBKG; NOD2; SRC; TNFRSF1A; RELA; CALM1;
Canonical NF-kappaB pathway	7	35	2.43056	4.3729	0.00087	1	NFKB1; PRKCA; IKBKG; NOD2; SRC; TNFRSF1A; RELA;
Sodium-coupled sulphate, di- and tri-carboxylate transporters	3	5	1.04167	13.1212	0.00089	1	SLC13A2; SLC13A3; SLC13A5;
S1P2 pathway	6	26	2.08333	5.04636	0.00093	1	GNAI1; GNAI3; GNAO1; S1PR2; FOS; JUN;
Regulation of Telomerase	10	68	3.47222	3.21444	0.00095	1	IL2; ESR1; TERT; NFKB1; FOS; JUN; HDAC1; RBBP4; RBBP7;
Endogenous TLR signaling	9	57	3.125	3.45158	0.00101	1	NFKB1; PRKCA; TLR2; IKBKG; NOD2; SRC; TLR4; TNFRSF1A;
Integrin-linked kinase signaling	47	654	16.3194	1.56982	0.00101	1	ABCG2; ARG1; CA9; IL2; IL5; IL6; ABCB1; PKN1; HRAS; VEGFA; NT5E; CREB1; ESR1; CAMK2B; MAPT; TERT; NFKB1; PRKCA; PRKCH; CDC25B; PLA2G4A; HK1; HK2; ACHE; DUSP1; EP300; FKBP4; FOS; GSK3B; JUN; PTPN7; HDAC1; SRC; HES1; KLK2; CAMK2A; MMP2; MMP9; NOS2; PTPN1; AR; NR3C1; NR1I3; RBBP4; RBBP7; RELA; CALM1;
TCR signaling in naïve CD4+ T cells	15	133	5.20833	2.46456	0.00104	1	IL2; HRAS; NFKB1; PRKCA; PLCG1; FOS; JUN; IKBKG; NOD2; PTPN7; SRC; PTPN6; TNFRSF1A; RELA; CALM1;
Signaling events regulated by Ret tyrosine kinase	10	69	3.47222	3.16786	0.00107	1	HRAS; CREB1; NFKB1; PRKCA; JUN; IKBKG; NOD2; SRC; TNFRSF1A; RELA;
G alpha (q) signalling events	14	120	4.86111	2.54956	0.00109	1	NMUR2; DAGLA; PRKCH; LPAR1; LPAR3; LPAR2; LPAR4; LPAR6; MGLL; HTR2B; HTR2C; PTGFR; GCGR; LTB4R;
Regulation of p38-alpha and p38-beta	17	164	5.90278	2.26505	0.00127	1	IL2; IL5; CREB1; ESR1; PRKCA; PRKCH; CDC25B; PLA2G4A; DUSP1; EP300; FOS; GSK3B; JUN; SRC; NOS2; PTPN1; CALM1;
Cytochrome P450 - arranged by substrate type	8	48	2.77778	3.64372	0.00134	1	CYP1B1; CYP2C8; CYP2A6; CYP19A1; CYP1A1; CYP24A1; CYP27B1; CYP17A1;
FGF signaling pathway	8	48	2.77778	3.64372	0.00134	1	FGF1; PLCG1; FOS; JUN; SRC; CAMK2A; MET; MMP9;
Hedgehog signaling events mediated by Gli proteins	8	48	2.77778	3.64372	0.00134	1	LGALS3; GNAI1; GNAI3; GNAO1; GSK3B; HDAC1; RBBP4; RBBP7;
CREB phosphorylation through the activation of Ras	6	28	2.08333	4.68603	0.0014	1	HRAS; CREB1; CAMK2B; PLA2G4A; CAMK2A; CALM1;
Regulation of cytoplasmic and nuclear SMAD2/3 signaling	26	305	9.02778	1.86239	0.00149	1	IL2; IL5; PKN1; CREB1; ESR1; CAMK2B; MAPT; PRKCA; PRKCH; CDC25B; PLA2G4A; DUSP1; EP300; FOS; GSK3B; JUN; HDAC1; SRC; CAMK2A; NOS2; PTPN1; AR; NR3C1; RBBP4;
TGF-beta receptor signaling	26	305	9.02778	1.86239	0.00149	1	IL2; IL5; PKN1; CREB1; ESR1; CAMK2B; MAPT; PRKCA; PRKCH; CDC25B; PLA2G4A; DUSP1; EP300; FOS; GSK3B; JUN; HDAC1; SRC; CAMK2A; NOS2; PTPN1; AR; NR3C1; RBBP4;
Regulation of nuclear SMAD2/3 signaling	26	305	9.02778	1.86239	0.00149	1	IL2; IL5; PKN1; CREB1; ESR1; CAMK2B; MAPT; PRKCA; PRKCH; CDC25B; PLA2G4A; DUSP1; EP300; FOS; GSK3B; JUN; HDAC1; SRC; CAMK2A; NOS2; PTPN1; AR; NR3C1; RBBP4;
Class A/1 (Rhodopsin-like receptors)	24	274	8.33333	1.91368	0.00157	1	NMUR2; OPRD1; ADORA1; CCR4; CNR1; CNR2; LPAR1; LPAR3; LPAR2; LPAR4; LPAR6; OXER1; PTGER2; S1PR2; ADORA3; HTR2B; HTR2C; ADORA2B; ADRB1; ADRB2;
Osteopontin-mediated events	6	29	2.08333	4.5245	0.0017	1	NFKB1; FOS; JUN; MMP2; MMP9; RELA;
S1P3 pathway	6	29	2.08333	4.5245	0.0017	1	VEGFA; GNAI1; GNAI3; GNAO1; S1PR2; SRC;

PLC beta mediated events	7	39	2.43056	3.92451	0.00171	1	CREB1; GNAI1; GNAI3; GNAO1; PRKCA; PLA2G4A; CALM1;
Cyclin B2 mediated events	3	6	1.04167	10.9379	0.00171	1	CDC25A; CDC25B; CDC25C;
Posttranslational regulation of adherens junction stability and dissassembly	21	231	7.29167	1.98627	0.00194	1	FGF1; HRAS; TERT; PLCG1; EP300; FKBP4; FOS; GSK3B; JUN; HDAC1; SRC; KLK2; CAMK2A; MET; MMP2; MMP9; PTPN1; PTPN6; AR; EGFR; LRP6;
G-protein mediated events	7	40	2.43056	3.82643	0.00199	1	CREB1; GNAI1; GNAI3; GNAO1; PRKCA; PLA2G4A; CALM1;
Serotonin clearance from the synaptic cleft	2	2	0.69444	21.8396	0.00209	1	MAOA; ALDH2;
Metabolism of serotonin	2	2	0.69444	21.8396	0.00209	1	MAOA; ALDH2;
Estrogen biosynthesis	2	2	0.69444	21.8396	0.00209	1	HSD17B1; CYP19A1;
Monoamines are oxidized to aldehydes by MAOA and MAOB, producing NH3 and	2	2	0.69444	21.8396	0.00209	1	MAOA; MAOB;
melatonin degradation II	2	2	0.69444	21.8396	0.00209	1	MAOA; MAOB;
G0 and Early G1	5	21	1.73611	5.20781	0.00216	1	DYRK1A; TOP2A; CDC25A; HDAC1; RBBP4;
IL2-mediated signaling events	13	115	4.51389	2.4705	0.00217	1	IL2; HRAS; TERT; NFKB1; PRKCA; FOS; JUN; IKBKG; NOD2; SRC; TNFRSF1A; RELA; CALM1;
Integrins in angiogenesis	9	64	3.125	3.07412	0.00234	1	FGF2; VEGFA; NFKB1; FOS; JUN; SRC; MMP2; MMP9; RELA;
Validated nuclear estrogen receptor alpha network	9	64	3.125	3.07412	0.00234	1	MPG; ESR1; ESR2; EP300; JUN; NDUFV3; HDAC1; PGR; NR0B2;
Aurora A signaling	9	64	3.125	3.07412	0.00234	1	NFKB1; PRKCA; CDC25B; GSK3B; IKBKG; NOD2; SRC; TNFRSF1A; RELA;
Syndecan-2-mediated signaling events	10	77	3.47222	2.83877	0.0025	1	FGF1; HRAS; PLCG1; FOS; JUN; SRC; CAMK2A; MET; MMP2; MMP9;
Ras signaling in the CD4+ TCR pathway	7	42	2.43056	3.64426	0.00266	1	IL2; HRAS; PRKCA; FOS; JUN; PTPN7; CALM1;
Purine salvage	3	7	1.04167	9.37761	0.0029	1	ADA; ADK; HPRT1;
Organic cation transport	3	7	1.04167	9.37761	0.0029	1	SLC22A1; SLC22A2; SLC22A3;
dopamine degradation	3	7	1.04167	9.37761	0.0029	1	MAOA; MAOB; ALDH2;
acetone degradation I (to methylglyoxal)	3	7	1.04167	9.37761	0.0029	1	AKR1B10; AKR1B1; ADH1B;
Glycogen breakdown (glycogenolysis)	4	14	1.38889	6.25101	0.00299	1	AGL; PYGB; PYGM; CALM1;
S1P4 pathway	4	14	1.38889	6.25101	0.00299	1	GNAI1; GNAI3; GNAO1; PLCG1;
ALK1 signaling events	26	321	9.02778	1.76956	0.00306	1	IL2; IL5; PKN1; CREB1; ESR1; CAMK2B; MAPT; PRKCA; PRKCH; CDC25B; PLA2G4A; DUSP1; EP300; FOS; GSK3B; JUN; HDAC1; SRC; CAMK2A; NOS2; PTPN1; AR; NR3C1; RBBP4;
p75(NTR)-mediated signaling	17	178	5.90278	2.08691	0.00308	1	HRAS; CREB1; NFKB1; DNM1; PRKCA; PLCG1; APP; FOS; GSK3B; IKBKG; NOD2; SRC; TRPV1; CAMK2A; TNFRSF1A;
BMP receptor signaling	20	226	6.94444	1.93358	0.00338	1	IL2; IL5; PKN1; CREB1; ESR1; CAMK2B; MAPT; PRKCA; PRKCH; CDC25B; PLA2G4A; DUSP1; EP300; FOS; GSK3B; JUN; SRC; NOS2; PTPN1; CALM1;

Root	ALK1 pathway	26	324	9.02778	1.75318	0.00347	1	IL2; IL5; PKN1; CREB1; ESR1; CAMK2B; MAPT; PRKCA; PRKCH; CDC25B; PLA2G4A; DUSP1; EP300; FOS; GSK3B; JUN; HDAC1; SRC; CAMK2A; NOS2; PTPN1; AR; NR3C1; RBBP4;
	CREB phosphorylation through the activation of CaMKII	4	15	1.38889	5.83455	0.00393	1	CREB1; CAMK2B; CAMK2A; CALM1;
	Eicosanoid ligand-binding receptors	4	15	1.38889	5.83455	0.00393	1	OXER1; PTGER2; PTGFR; LTB4R;
	Activation of NMDA receptor upon glutamate binding and postsynaptic events	6	34	2.08333	3.85933	0.00398	1	HRAS; CREB1; CAMK2B; PLA2G4A; CAMK2A; CALM1;
	Post NMDA receptor activation events	6	34	2.08333	3.85933	0.00398	1	HRAS; CREB1; CAMK2B; PLA2G4A; CAMK2A; CALM1;
	Purine metabolism	5	24	1.73611	4.55711	0.00403	1	XDH; NT5E; ADA; ADK; HPRT1;
	androgen biosynthesis	3	8	1.04167	8.20687	0.00448	1	HSD17B3; AKR1C3; CYP17A1;
	S1P5 pathway	3	8	1.04167	8.20687	0.00448	1	GNAI1; GNAI3; GNAO1;
	CD40/CD40L signaling	8	58	2.77778	3.0156	0.00457	1	NFKB1; PRKCA; JUN; IKBKG; NOD2; SRC; TNFRSF1A; RELA;
	IL12-mediated signaling events	12	111	4.16667	2.36279	0.00459	1	IL2; IL6; NFKB1; PRKCA; FOS; JUN; IKBKG; NOD2; SRC; NOS2; TNFRSF1A; RELA;
	Signal amplification	4	16	1.38889	5.47012	0.00506	1	GNAI1; GNAI3; PLA2G4A; SRC;
	Glucose metabolism	6	36	2.08333	3.64498	0.00533	1	AGL; PYGB; PYGM; MDH1; MDH2; CALM1;
	Neuronal System	17	188	5.90278	1.97591	0.0054	1	MAOA; KCND3; HRAS; CREB1; CAMK2B; GNAI1; GNAI3; PLA2G4A; KCNK2; SLC22A2; ACHE; SLC6A11; SLC6A13; CAMK2A; GLUL; ALDH2; CALM1;
	GPCR ligand binding	26	336	9.02778	1.69057	0.00563	1	NMUR2; OPRD1; ADORA1; CCR4; CNR1; CNR2; LPAR1; LPAR3; LPAR2; LPAR4; LPAR6; OXER1; PTGER2; S1PR2; ADORA3; HTR2B; HTR2C; ADORA2B; ADRB1; ADRB2; ADRB3; FFAR4; PTGFR; GCGR; ADCYAP1R1; LTB4R;
	Arachidonate production from DAG	2	3	0.69444	14.5839	0.00608	1	DAGLA; MGLL;
	Na+-dependent glucose transporters	2	3	0.69444	14.5839	0.00608	1	SLC5A1; SLC5A2;
	methylglyoxal degradation III	2	3	0.69444	14.5839	0.00608	1	AKR1B10; AKR1B1;
	CXCR3-mediated signaling	6	37	2.08333	3.54649	0.00613	1	HRAS; DNM1; GNAI1; GNAI3; GNAO1; SRC;
	Ras activation uopn Ca2+ infux through NMDA receptor	4	17	1.38889	5.14854	0.00638	1	HRAS; CAMK2B; CAMK2A; CALM1;
	Adrenoceptors	3	9	1.04167	7.29601	0.00649	1	ADRB1; ADRB2; ADRB3;
	adenosine nucleotides degradation II	3	9	1.04167	7.29601	0.00649	1	XDH; NT5E; ADA;
	Trk receptor signaling mediated by PI3K and PLC-gamma	8	63	2.77778	2.7763	0.00758	1	HRAS; CREB1; PLCG1; FOS; GSK3B; SRC; TRPV1; CAMK2A;
	Validated transcriptional targets of AP1 family members Fra1	13	134	4.51389	2.12023	0.00808	1	IL2; IL5; IL6; HRAS; PRKCA; EP300; FOS; JUN; PTPN7; MMP2; MMP9; PTPN1; CALM1;

Base-free sugar-phosphate removal via the single-nucleotide replacement pathway	3	10	1.04167	6.56714	0.00897	1	MPG; APEX1; POLB;
Signaling events mediated by the Hedgehog family	8	65	2.77778	2.69089	0.00913	1	LGALS3; GNAI1; GNAI3; GNAO1; GSK3B; HDAC1; RBBP4; RBBP7;
EGFR interacts with phospholipase C-gamma	5	29	1.73611	3.77167	0.00934	1	CREB1; PRKCA; PLCG1; EGFR; CALM1;
VEGFR1 specific signals	5	29	1.73611	3.77167	0.00934	1	PGF; VEGFA; PRKCA; PLCG1; CALM1;
Opioid Signalling	7	53	2.43056	2.88805	0.00991	1	CREB1; GNAI1; GNAI3; GNAO1; PRKCA; PLA2G4A; CALM1;
BCR signaling pathway	8	67	2.77778	2.61058	0.01091	1	HRAS; NFKB1; FOS; JUN; IKBKG; PTPN6; RELA; CALM1;
NFkB and MAP kinases activation mediated by TLR4 signaling repertoire	7	54	2.43056	2.83457	0.01095	1	CREB1; NFKB1; FOS; JUN; IKBKG; TLR4; RELA;
Role of Calcineurin-dependent NFAT signaling in lymphocytes	10	95	3.47222	2.30096	0.01117	1	IL2; IL5; PRKCA; PRKCH; EP300; FOS; GSK3B; JUN; PTPN1; CALM1;
FOXA transcription factor networks	9	81	3.125	2.42901	0.01126	1	CREB1; ESR1; TTR; EP300; FOS; JUN; NDUFV3; AR; NR3C1;
Cholesterol biosynthesis	4	20	1.38889	4.37664	0.01166	1	LSS; HMGCR; DHCR7; EBP;
Reuptake of GABA	2	4	0.69444	10.947	0.01179	1	SLC6A11; SLC6A13;
Ethanol oxidation	2	4	0.69444	10.947	0.01179	1	ALDH1A1; ALDH2;
Proton-coupled monocarboxylate transport	2	4	0.69444	10.947	0.01179	1	SLC16A7; SLC16A3;
trans,trans-farnesyl diphosphate biosynthesis	2	4	0.69444	10.947	0.01179	1	FDPS; GGPS1;
adenine and adenosine salvage	2	4	0.69444	10.947	0.01179	1	ADA; HPRT1;
TAK1 activates NFkB by phosphorylation and activation of IKKs complex	3	11	1.04167	5.97067	0.01192	1	NFKB1; IKBKG; RELA;
Signaling by Aurora kinases	10	98	3.47222	2.23053	0.01374	1	NFKB1; PRKCA; CDC25B; GSK3B; IKBKG; NOD2; SRC; TNFRSF1A; MYLK; RELA;
Regulation of CDC42 activity	48	768	16.6667	1.36524	0.01398	1	ABCG2; ARG1; CA9; IL2; IL5; IL6; ABCB1; PKN1; HRAS; VEGFA; NT5E; CREB1; ESR1; CAMK2B; MAPT; TERT; NFKB1; PRKCA; PRKCH; CDC25B; PLA2G4A; HK1; HK2; ACHE; DUSP1; PLCG1; EP300; FKBP4; FOS; GSK3B; JUN; PTPN7; HDAC1; SRC; HES1; KLK2; CAMK2A; MMP2; MMP9; NOS2; PTPN1; AR; NR3C1; NR1I3; RBBP4; RBBP7; RELA; CALM1;
Stabilization and expansion of the E-cadherin adherens junction	21	275	7.29167	1.66848	0.01416	1	FGF1; HRAS; TERT; PLCG1; EP300; FKBP4; FOS; GSK3B; JUN; HDAC1; SRC; KLK2; CAMK2A; MET; MMP2; MMP9; PTPN1; PTPN6; AR; EGFR; LRP6;
E-cadherin signaling in the nascent adherens junction	21	275	7.29167	1.66848	0.01416	1	FGF1; HRAS; TERT; PLCG1; EP300; FKBP4; FOS; GSK3B; JUN; HDAC1; SRC; KLK2; CAMK2A; MET; MMP2; MMP9; PTPN1; PTPN6; AR; EGFR; LRP6;

FOXA1 transcription factor network	6	44	2.08333	2.98241	0.01425	1	ESR1; EP300; FOS; JUN; NDUFV3; AR;
Resolution of AP sites via the single-nucleotide replacement pathway	3	12	1.04167	5.47353	0.01536	1	MPG; APEX1; POLB;
Post-chaperonin tubulin folding pathway	3	12	1.04167	5.47353	0.01536	1	TUBB1; TUBB2B; TUBB3;
purine nucleotides degradation II (aerobic)	3	12	1.04167	5.47353	0.01536	1	XDH; NT5E; ADA;
MyD88-independent cascade initiated on plasma membrane	7	58	2.43056	2.63912	0.01592	1	CREB1; NFKB1; FOS; JUN; IKBKG; TLR4; RELA;
CDC42 signaling events	47	755	16.3194	1.35982	0.01605	1	ABCG2; ARG1; CA9; IL2; IL5; IL6; ABCB1; PKN1; HRAS; VEGFA; NT5E; CREB1; ESR1; CAMK2B; MAPT; TERT; NFKB1; PRKCA; PRKCH; CDC25B; PLA2G4A; HK1; HK2; ACHE; DUSP1; EP300; FKBP4; FOS; GSK3B; JUN; PTPN7; HDAC1; SRC; HES1; KLK2; CAMK2A; MMP2; MMP9; NOS2; PTPN1; AR; NR3C1; NR1I3; RBBP4; RBBP7; RELA; CALM1;
E-cadherin signaling events	21	280	7.29167	1.63869	0.01705	1	FGF1; HRAS; TERT; PLCG1; EP300; FKBP4; FOS; GSK3B; JUN; HDAC1; SRC; KLK2; CAMK2A; MET; MMP2; MMP9; PTPN1; PTPN6; AR; EGFR; LRP6;
SLC-mediated transmembrane transport	19	247	6.59722	1.68078	0.01786	1	SLC16A7; SLC28A3; SLC5A1; SLC5A2; SLC22A1; SLC22A2; SLC22A6; SLC22A8; HK1; HK2; SLC22A3; SLC6A11; SLC6A13; TPR; SLC16A3; SLC37A4; SLC13A2; SLC13A3; SLC13A5;
Activation of CaMK IV	2	5	0.69444	8.76198	0.01906	1	CREB1; CALM1;
CaMK IV-mediated phosphorylation of CREB	2	5	0.69444	8.76198	0.01906	1	CREB1; CALM1;
Glucocorticoid biosynthesis	2	5	0.69444	8.76198	0.01906	1	HSD11B1; CYP17A1;
Organic anion transport	2	5	0.69444	8.76198	0.01906	1	SLC22A6; SLC22A8;
putrescine degradation III	2	5	0.69444	8.76198	0.01906	1	MAOB; ALDH2;
aspartate degradation II	2	5	0.69444	8.76198	0.01906	1	MDH1; MDH2;
superpathway of melatonin degradation	2	5	0.69444	8.76198	0.01906	1	MAOA; MAOB;
G-protein activation	3	13	1.04167	5.05281	0.0193	1	GNAI1; GNAI3; GNAO1;
Viral dsRNA:TLR3:TRIF Complex Activates RIP1	3	13	1.04167	5.05281	0.0193	1	NFKB1; IKBKG; RELA;
TRAF6 mediated NF-kB	3	13	1.04167	5.05281	0.0193	1	NFKB1; IKBKG; RELA;
superpathway of geranylgeranyldiphosphate biosynthesis I (via mevalonate)	3	13	1.04167	5.05281	0.0193	1	FDPS; GGPS1; HMGCR;
cholesterol biosynthesis II (via 24,25-dihydrolanosterol)	3	13	1.04167	5.05281	0.0193	1	LSS; DHCR7; EBP;
cholesterol biosynthesis I	3	13	1.04167	5.05281	0.0193	1	LSS; DHCR7; EBP;

cholesterol biosynthesis III (via desmosterol)	3	13	1.04167	5.05281	0.0193	1	LSS; DHCR7; EBP;
DNA-PK pathway in nonhomologous end joining	3	13	1.04167	5.05281	0.0193	1	POLL; POLM; DNTT;
Retinoic acid receptors-mediated signaling	6	47	2.08333	2.79208	0.01934	1	PRKCA; EP300; HDAC1; RARA; RARB; RARG;
PLK1 signaling events	10	104	3.47222	2.10185	0.02016	1	NFKB1; PRKCA; CDC25B; CDC25C; GSK3B; IKBKG; NOD2; SRC; TNFRSF1A; RELA;
Toll Receptor Cascades	9	90	3.125	2.18614	0.02135	1	CREB1; NFKB1; TLR2; FOS; JUN; IKBKG; TLR4; TLR9; RELA;
Amine ligand-binding receptors	5	36	1.73611	3.03849	0.02286	1	HTR2B; HTR2C; ADRB1; ADRB2; ADRB3;
Calcineurin-regulated NFAT-dependent transcription in lymphocytes	6	49	2.08333	2.67814	0.02335	1	IL2; IL5; FOS; JUN; PTPN1; CALM1;
Cyclin A/B1 associated events during G2/M transition	3	14	1.04167	4.69215	0.02375	1	CDC25A; CDC25B; CDC25C;
Xenobiotics	3	14	1.04167	4.69215	0.02375	1	CYP2C8; CYP2A6; CYP1A1;
Atypical NF-kappaB pathway	3	14	1.04167	4.69215	0.02375	1	NFKB1; SRC; RELA;
Platelet activation, signaling and aggregation	12	138	4.16667	1.90054	0.02378	1	DAGLA; GNAI1; GNAI3; PRKCA; PRKCH; MGLL; PLA2G4A; HSPA5; SRC; PTPN1; TUBA4A; CALM1;
Signaling events mediated by TCPTP	9	92	3.125	2.13862	0.02428	1	ARG1; IL5; ALOX15; VEGFA; SRC; MET; PTPN1; PTPN6; EGFR;
Regulation of Androgen receptor activity	10	108	3.47222	2.02402	0.02552	1	PKN1; EP300; FKBP4; GSK3B; JUN; HDAC1; SRC; KLK2; AR; NR3C1;
TRAF6 Mediated Induction of proinflammatory cytokines	6	50	2.08333	2.62459	0.02555	1	CREB1; NFKB1; FOS; JUN; IKBKG; RELA;
Signaling mediated by p38-alpha and p38-beta	6	50	2.08333	2.62459	0.02555	1	CREB1; ESR1; CDC25B; PLA2G4A; JUN; NOS2;
Toll Like Receptor TLR1:TLR2 Cascade	7	64	2.43056	2.39174	0.02612	1	CREB1; NFKB1; TLR2; FOS; JUN; IKBKG; RELA;
Toll Like Receptor TLR6:TLR2 Cascade	7	64	2.43056	2.39174	0.02612	1	CREB1; NFKB1; TLR2; FOS; JUN; IKBKG; RELA;
Validated transcriptional targets of deltaNp63 isoforms	7	64	2.43056	2.39174	0.02612	1	FASN; TOP2A; AXL; ADA; GSK3B; HES1; LRP6;
Glypican 1 network	73	1296	25.3472	1.23032	0.02687	1	ABCG2; ARG1; CA9; FGF1; FGF2; IL2; IL5; IL6; LGALS1; ALOX15; ABCB1; ABCC1; PKN1; HRAS; VEGFA; NT5E; CREB1; ESR1; CAMK2B; MAPT; TERT; NFKB1; DNM1; GNAI1; GNAI3; GNAO1; PRKCA; PRKCH; CDC25A; CDC25B; PLA2G4A; PLA2G2A; DNTT; HK1; HK2; PDCD4; ACHE; DUSP1; PLCG1; APP; ADRB2; EP300; FKBP4; FOS; GSK3B; JUN; MMP12; NDUFAF2; IKBKG; NOD2; PTPN7; HDAC1; SRC; ODC1; HES1; KLK2; CAMK2A; MET; MMP2; MMP9; NOS2; PTPN1; PTPN6;

Polo-like kinase signaling events in the cell cycle	10	109	3.47222	2.00545	0.02701	1	NFKB1; PRKCA; CDC25B; CDC25C; GSK3B; IKBKG; NOD2; SRC; TNFRSF1A; RELA;
ADP signalling through P2Y purinoceptor 12	2	6	0.69444	7.30408	0.02773	1	GNAI1; GNAI3;
CREB phosphorylation through the activation of CaMKK	2	6	0.69444	7.30408	0.02773	1	CREB1; CALM1;
FGFR1b ligand binding and activation	2	6	0.69444	7.30408	0.02773	1	FGF1; FGF2;
Vitamins	2	6	0.69444	7.30408	0.02773	1	CYP24A1; CYP27B1;
Interleukin-1 processing	2	6	0.69444	7.30408	0.02773	1	NFKB1; RELA;
ethanol degradation II (cytosol)	2	6	0.69444	7.30408	0.02773	1	ADH1B; ALDH2;
Toll Like Receptor 9 (TLR9) Cascade	7	65	2.43056	2.35495	0.02816	1	CREB1; NFKB1; FOS; JUN; IKBKG; TLR9; RELA;
Toll Like Receptor 2 (TLR2) Cascade	7	65	2.43056	2.35495	0.02816	1	CREB1; NFKB1; TLR2; FOS; JUN; IKBKG; RELA;
Downstream signaling in naïve CD8+ T cells	7	65	2.43056	2.35495	0.02816	1	IL2; HRAS; PRKCA; FOS; JUN; PTPN7; CALM1;
Endogenous sterols	3	15	1.04167	4.37955	0.0287	1	CYP1B1; CYP19A1; CYP17A1;
Neurotransmitter Release Cycle	4	26	1.38889	3.36704	0.02904	1	MAOA; SLC22A2; SLC6A11; SLC6A13;
Signaling by FGFR	9	95	3.125	2.07109	0.02918	1	FGF1; FGF2; HRAS; CREB1; PRKCA; PLCG1; SRC; EGFR;
Phospholipase C-mediated cascade	4	27	1.38889	3.24238	0.0329	1	CREB1; PRKCA; PLCG1; CALM1;
DAG and IP3 signaling	4	27	1.38889	3.24238	0.0329	1	CREB1; PRKCA; PLCG1; CALM1;
Ca-dependent events	4	27	1.38889	3.24238	0.0329	1	CREB1; PRKCA; PLA2G4A; CALM1;
Hexose transport	5	40	1.73611	2.73472	0.03444	1	SLC5A1; HK1; HK2; TPR; SLC37A4;
amb2 Integrin signaling	5	40	1.73611	2.73472	0.03444	1	IL6; NFKB1; SRC; MMP2; MMP9;
KitReceptor	6	54	2.08333	2.43021	0.03572	1	HRAS; PRKCA; PLCG1; EP300; SRC; PTPN6;
PECAM1 interactions	2	7	0.69444	6.26213	0.03766	1	PLCG1; PTPN6;
FGFR1 ligand binding and activation	2	7	0.69444	6.26213	0.03766	1	FGF1; FGF2;
Vitamin A uptake in enterocytes	2	7	0.69444	6.26213	0.03766	1	PLB1; PNLIP;
Vitamin D (calciferol)	2	7	0.69444	6.26213	0.03766	1	CYP24A1; CYP27B1;
Interleukin receptor SHC	2	7	0.69444	6.26213	0.03766	1	HRAS; PTPN6;
Removal of DNA patch containing abasic residue	3	17	1.04167	3.86461	0.04011	1	MPG; APEX1; POLB;
Resolution of AP sites via the multiple-nucleotide patch replacement pathway	3	17	1.04167	3.86461	0.04011	1	MPG; APEX1; POLB;
Inflammasomes	3	17	1.04167	3.86461	0.04011	1	NFKB1; APP; RELA;
Neurotrophic factor-mediated Trk receptor signaling	9	101	3.125	1.94807	0.041	1	HRAS; CREB1; DNM1; PLCG1; FOS; GSK3B; SRC; TRPV1; CAMK2A;
FOXM1 transcription factor network	5	42	1.73611	2.60453	0.04138	1	ESR1; CDC25B; EP300; FOS; MMP2;

	Calcium signaling in the CD4+ TCR pathway	4	29	1.38889	3.01884	0.04148	1	IL2; FOS; JUN; CALM1;
	TRIF mediated TLR3 signaling	6	56	2.08333	2.34343	0.04166	1	CREB1; NFKB1; FOS; JUN; IKBKG; RELA;
	Toll Like Receptor 3 (TLR3) Cascade	6	56	2.08333	2.34343	0.04166	1	CREB1; NFKB1; FOS; JUN; IKBKG; RELA;
	Nucleotide-binding domain, leucine rich repeat containing receptor (NLR) signaling pathways	5	43	1.73611	2.54397	0.04514	1	NFKB1; APP; IKBKG; NOD2; RELA;
	Activated TLR4 signalling	7	72	2.43056	2.12603	0.0456	1	CREB1; NFKB1; FOS; JUN; IKBKG; TLR4; RELA;
	Translesion synthesis by Pol eta	1	1	0.34722	21.8396	0.04579	1	POLH;
	glutamine biosynthesis I	1	1	0.34722	21.8396	0.04579	1	GLUL;
	1,25-dihydroxyvitamin D3 biosynthesis	1	1	0.34722	21.8396	0.04579	1	CYP27B1;
	lanosterol biosynthesis	1	1	0.34722	21.8396	0.04579	1	LSS;
	adenine and adenosine salvage	1	1	0.34722	21.8396	0.04579	1	ADK;
	L-dopachrome biosynthesis	1	1	0.34722	21.8396	0.04579	1	TYR;
	PLC-gamma1 signalling	4	30	1.38889	2.91825	0.0462	1	CREB1; PRKCA; PLCG1; CALM1;
	Na+/Cl- dependent neurotransmitter transporters	3	18	1.04167	3.65003	0.04655	1	SLC22A2; SLC6A11; SLC6A13;
	IFN-gamma pathway	71	1293	24.6528	1.19939	0.04802	1	ABCG2; ARG1; CA9; DAPK1; FGF1; IL2; IL5; IL6; LGALS1; ALOX15; ABCB1; ABCC1; PKN1; HRAS; VEGFA; NT5E; CREB1; ESR1; CAMK2B; MAPT; TERT; NFKB1; DNM1; GNAI1; GNAI3; GNAO1; PRKCA; PRKCH; CDC25A; CDC25B; PLA2G4A; DNTT; HK1; HK2; PDCD4; ACHE; DUSP1; PLCG1; ADRB2; EP300; FKBP4; FOS; GSK3B; JUN; MMP12; NDUFAF2; IKBKG; NOD2; PTPN7; HDAC1; SRC; ODC1; HES1; KLK2; CAMK2A; MET; MMP2; MMP9; NOS2; PTPN1; PTPN6; TNFRSF1A; AR; NR3C1;
	Metabolism of nucleotides	6	58	2.08333	2.26264	0.04819	1	XDH; NT5E; ADA; ADK; CDA; HPRT1;
	Platelet sensitization by LDL	2	8	0.69444	5.48034	0.04872	1	PLA2G4A; PTPN6;
	Androgen biosynthesis	2	8	0.69444	5.48034	0.04872	1	HSD17B3; CYP17A1;
	Stabilization of mRNA by HuR	2	8	0.69444	5.48034	0.04872	1	ELAVL1; PRKCA;
	Glypican pathway	73	1335	25.3472	1.19438	0.04876	1	ABCG2; ARG1; CA9; FGF1; FGF2; IL2; IL5; IL6; LGALS1; ALOX15; ABCB1; ABCC1; PKN1; HRAS; VEGFA; NT5E; CREB1; ESR1; CAMK2B; MAPT; TERT; NFKB1; DNM1; GNAI1; GNAI3; GNAO1; PRKCA; PRKCH; CDC25A; CDC25B; PLA2G4A; PLA2G2A; DNTT; HK1; HK2; PDCD4; ACHE; DUSP1; PLCG1; APP; ADRB2; EP300; FKBP4; FOS; GSK3B; JUN; MMP12; NDUFAF2; IKBKG; NOD2; PTPN7; HDAC1; SRC; ODC1; HES1; KLK2; CAMK2A; MET; MMP2; MMP9; NOS2; PTPN1; PTPN6;
