# Supplementary figures and images for: Analysis of Bioactive Components in the Fruit, Roots, and Leaves of Alpinia oxyphylla by UPLC-MS/MS
Source: Evid Based Complement Alternat Med. 2021 Jul 9;2021:5592518. doi: 10.1155/2021/5592518 (PMC8286198; doi:10.1155/2021/5592518)

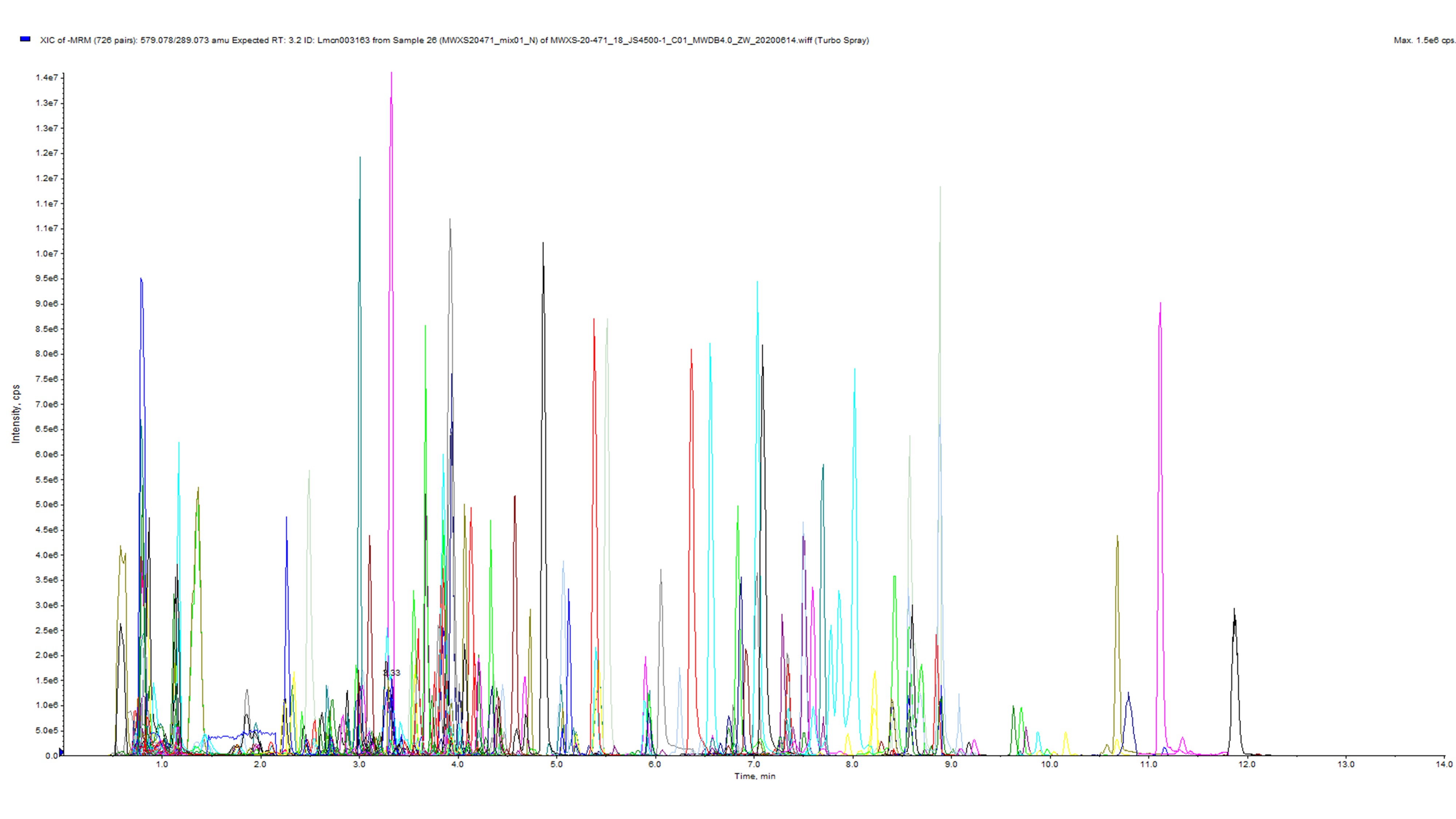

Supplement: Supplementary Materials — Figure S1: MRM metabolite detection. The multipeak diagram shows the substances that were detected in the sample, and each mass spectral peak with different colors represents one detected metabolite. Figure S2: OPLS-DA analysis model verification diagram. Table S1: 312 identified metabolites. Table S2: the target genes were enriched in multiple pathways. [file 5592518.f1.zip › 5592518.f1/Figure S1.jpg]

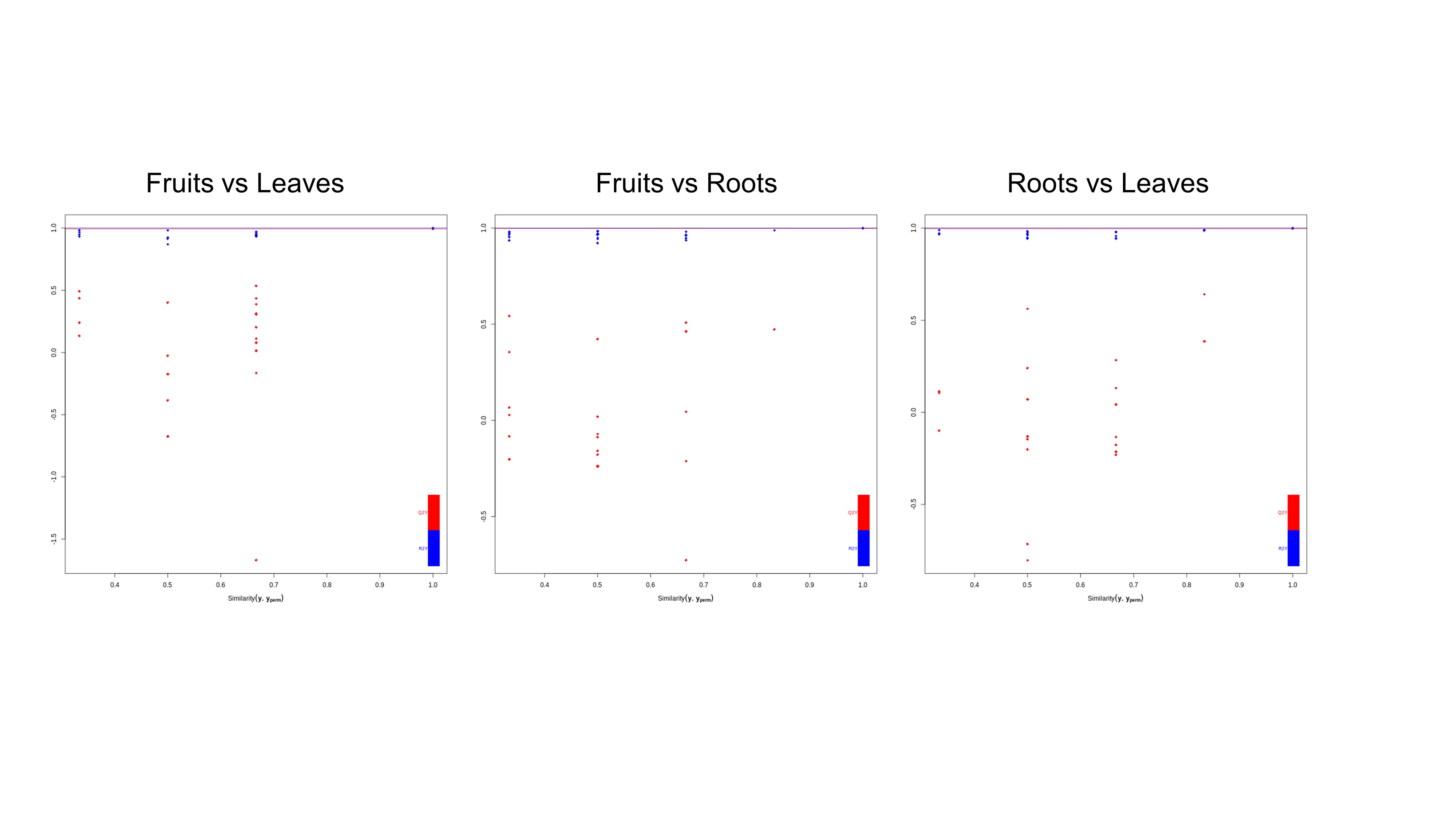

Supplement: Supplementary Materials — Figure S1: MRM metabolite detection. The multipeak diagram shows the substances that were detected in the sample, and each mass spectral peak with different colors represents one detected metabolite. Figure S2: OPLS-DA analysis model verification diagram. Table S1: 312 identified metabolites. Table S2: the target genes were enriched in multiple pathways. [file 5592518.f1.zip › 5592518.f1/Figure S2.jpg]
